# Experimental Investigation of Different Fineness and Firing Temperatures on Pellets Properties of Different Iron Ore fines from Indian Mines

**DOI:** 10.3390/ma15124220

**Published:** 2022-06-14

**Authors:** Rakesh Prasad, Shatrughan Soren, L. A. Kumaraswamidhas, Chandan Pandey, S. K. Pan

**Affiliations:** 1 Fuel, Minerals and Metallurgical Engineering Department, Indian Institute of Technology Dhanbad, Dhanbad 826004, India; ssoren@iitism.ac.in; 2 Mechanical Engineering Department, Hindustan College of Science & Technology, Mathura 281122, India; 3 Mining and Machinery Engineering Department, Indian Institute of Technology Dhanbad, Dhanbad 826004, India; akdhas1978@iitism.ac.in; 4 Mechanical Engineering Department, Indian Institute of Technology Jodhpur, Karwar 342037, India; 5 RDCIS-Ranchi, Ranchi 826004, India; panswapan1959@gmail.com

**Keywords:** ore fines, pelletizing, composite agglomerate, C.C.S., A.P., microstructure, thermal kinetics

## Abstract

In India, during mining and ore processing, ore fine generation is a common phenomenon, in which more than 60% of process ore becomes discarded material. To explore the alternative of high-grade ores, mutual replacement with the utility of dump ore fines is the best way. With this perspective, Kiruburu iron ore mine (Iron Ore No.1) and Meghataburu iron ore mine (Iron Ore No.2) dumped fines were chosen for a Blaine no. investigation, in the connection of firing temperatures, to get optimum desirable physical properties, Cold Compression Strength (C.C.S.),and Apparent Porosity (A.P.), with physico-chemical properties, Reducibility Degradation Index (R.D.I.), and Reducibility Index (R.I.). To characterize pellet properties with input variables, a microstructure phase study has been conducted using a scanning electron microscope (S.E.M.), energy dispersive spectroscopy (EDS), and X-ray diffraction analysis (XRD). The Iron Ore No.1 and 2 fine pellets survey showed good, desirable properties, at the Blaine no., of 1678 cm^2^/g and 2311 cm^2^/g (corresponding to 200 mesh size), and the best results are attained at a firing temperature of 1300 °C. Thermal kinetic analysis of the heating of pellets has been done to knowthe activation energy of different ore characteristics. The results showed that Iron Ore No.2 pellets have high activation energy.

## 1. Introduction

Iron and steel are the most common commodities globally. Its consumption is ever increasing, at a very high rate, in developing countries such as India. The extraction of iron from ore through the blast furnace route is a very common and widely used technique all over the world. The feedstock of the blast furnace mainly contains lump ore, partial pellets, and sinter. Ore particles of various sizes, in a certain range, are suitable for feedstock to enhance the effectiveness of the process. Iron ore availability in India is mainly in the form of hematite, magnetite, and goethite. Mainly, hematite iron ore is found abundantly, and to use iron ore blast furnace feedstock, lump ore preparation is required. During the lump ore preparation, ore dressing generates a significant amount of fines. Washing generates micro fines (less than 30 μm), and approximately 60% of the ore fines are generated.

Along with the fine size, chemical components, such as alumina, cause higher flux consumption and the production of viscous content. Alumina and silica gangue content restricted the utility of iron fines, even when they were bearing enough iron. Moreover, hydroxyl bearing (goethite ore) content has high loss on ignition (LOI) in the iron ore concentrate. Therefore, these fines cannot employ in the furnace without property customization of feedstock, such as sintering and pelletization. Fineness of less than 0.5 mm hinders bed permeability, and high gangue content restricts the utility of fines. Pelletization has the freedom to use fines in different proportions of variable size ranges. Pellet feed chemistry can be altered, as per ores, so pelletization is, therefore, the only process of alternative use for dumped fines.

Dumped fines are collected for study to suggest an alternative to high-grade ore. During the ore’s processing, the generation of ore fines from lump ore is also an issue. Therefore, the utility of low-grade ore fines is supposed to be to produce feedstock for the blast furnace. The agglomerate should have optimum values of physical and physicochemical properties for effective and economic blast furnace operations [1]. Therefore, it is important to select optimum Blaine fineness for the production of good quality pellets [2].

Green pellets are forwarded for thermal treatment, where a certain temperature at which crystal transformation occurs is attained. Since oxides and gangue content reactions take place, silicate/slag phases, within the pellets, are formed. Therefore, sustainability of the bonding phases depends on microstructure formation, which is mainly governed by additives induced in the ore. Thermal treatment of pellets by oxidation is a phenomenon in the pelletization process. Heat hardening of pellets occurs due to the crystallization of iron oxides and silicate phases [3]. Moreover, hematite pellets do not exhibit satisfactory roasting during heating, so a temperature higher than 1300 °C is needed. Therefore, attainment of a high temperature is essential to execute metallurgical operations. To get adequate bonding phases, higher roasting with a narrower firing range is recommended. In this context, carbon composed pellets of hematite ore fines favor the optimal firing temperature [4]. Hence, the firing of pellets has a critical and decisive role in the properties of pellets. The selection of optimum firing temperature depends on the chemistry of the ore, the additive flux, and ore fineness [4,5].

The low-grade ore fines’ utility is for iron extraction, which is not allowed through conventional techniques. Due to the pre-treatment of pellet feed, having complex mineralogy and chemistry of the ore concentrate, few unconventional approaches are being employed to get good quality agglomerates. Although beneficiation of ore fines is the solution to this problem, iron recovery, however, is another concern. There are two approaches—reduction roasting and microwave-assisted heating—that are chiefly used to get optimal qualities of agglomerates. (a) Reduction roasting is the process in which higher iron oxides are reduced to low order oxides in the magnetite phase. The heat obtained causes fragmentations of ores to help liberate iron. In the aspect of the physical separation of iron, this method is good in yield with poor mineralogical constraints. Iron recovery from these fines, from 72% to 79%, may be obtained from 60% to 66% of iron-bearing oxide fines [6]. (b) Microwave heating promotes the thermal phase transformation of hematite ores to magnetite in the presence of carbon. As compared to conventional reduction roasting, microwaves accelerate the kinetics even with low-grade iron ores. Fe recovery up to 80%, from an iron grade of 57% iron, was seen with microwave roasting [7].

In the present work, there are two kinds of ores. One is taken from the Kiruburu iron ore mines (Iron Ore No.1), which is one of the captive iron ore mines of Steel Authority of India Limited (SAIL). Iron ore is being mined and processed at the rate of 4.25 MT/Annum. The second ore is taken from Meghataburu iron ore mines (Iron Ore No.2), running with an annual capacity of 6.5 million tons, which are severely affected by dump fines. Slope failure in the overburden dump threatens the mines and may bring the chance of interruption in production [8].

Iron Ore No.1 and No.2 fines have been investigated for different Blaine fineness and firing temperatures during heat hardening. Blaine’s fineness role in making good quality pellets, in conjunction with the firing temperature, has been investigated by many authors and researchers. None of them performed experiments with ore concentrate, with 400 mesh, and with firing temperatures up to 1350 °C. Since chosen ore fines are chiefly hematite base ore, they have poor roasting characteristics. Hence, to observe crystal formation/transformation, prepared pellets were fired up to 1350 °C, and a detailed microstructure analysis was done.

The microstructural investigation is an informative tool to control pellet quality based on the process parameters. A comparative study has been done on fineness, as well as the drying and heating processes. As per the chemistry of an ore pellet’s microstructure, the formation and impact on physical and physicochemical properties were investigated [9,10].

## 2. Experimental Work

### 2.1. Pellets Making

Iron ore concentrates were collected from Iron ore No.1 and Iron ore No.2, located in India. These ores are of pre-Cambrian origin, but Iron ore No.1 has a hard laminated nature, while Iron ore No.2 ore has a soft laminated nature.After, the concentration of ore is 62.42% Fe, 2.98% SiO_2_, 1.91% Al_2_O_3_, and 3.61% L.O.I. (Loss on Ignition). The chemistry of Iron Ore No.1 ore fines and the63.48% Fe, 3.07% SiO_2_, 2.68% Al_2_O_3_, and 4.52% L.O.I. of Iron Ore No.2 ore fines was observed.

#### 2.1.1. Fines Sample Preparation

This study is focused on ore fineness; therefore, the surface area is the key parameter of granulometry with the size and shape of particles. Thus, fines’ concentrated ground, for obtaining four levels of Blaine fineness, corresponds to four mesh (100, 200, 300 and 400) fineness.

Particle orientation leads to a variety of crystalline phase formations. Blaine fineness of ores was prepared, and their particle size analysis is done [11]. Table 1 (Iron Ore No.1 fines) and Table 2 (Iron Ore No.2 fines) show the fines size analysis concerning the available Blaine number.

#### 2.1.2. Green Pellets Preparation

The alkalinity of pellets is a desirable feature, and the addition of the alkaline compound is more advantageous in improving mechanical and metallurgical properties [12]. To encounter the acidic gangue (SiO_2_% + Al_2_O_3_%) content in Iron Ore No.1 and No.2 fines, flux hasbeen added to the ore fines. The raw material mix was composed of coke (−0.074 mm, 1% by weight); dolomite flux (−0.021 mm) to fix basicity 0.6; bentonite (−75 μm, 0.5% by weight) as a binder. Both ores were prepared with the same additives and binder in the same proportion of inclusion. Green pellets were prepared with an average size ranging from 8 to 16 mm.

### 2.2. Pellets Heat Hardening

#### Thermal Treatment Cycle

**Figure 1:**
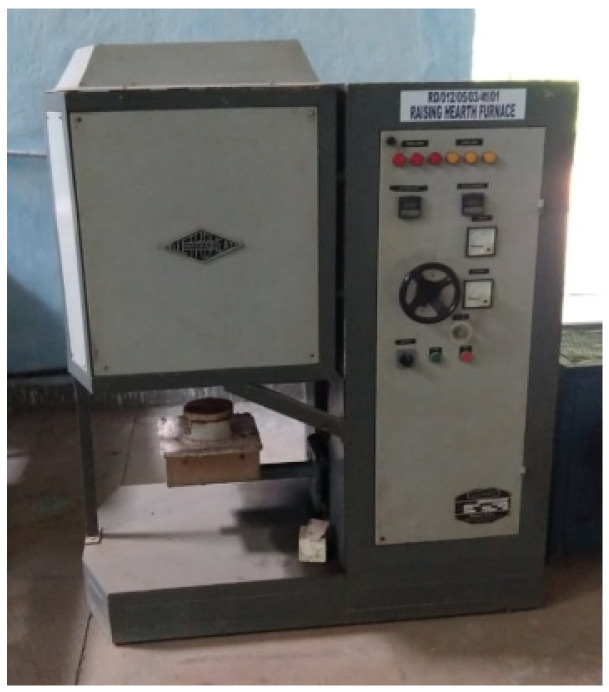
Raising Hearth Furnace (RHF).

The thermal treatment has great importance regarding pellet quality. Moreover, additive flux and binders become reactive during heat application and render the pellet’s structural strength. Heat hardenings of green samples were carried out in a raising hearth furnace (RHF), as shown in Figure 1. The furnace is equipped with a single chamber and electrical element, with a maximum loading capacity up to 5 Kg samples at one time in a ceramic cylindrical jar.

The process of pellet heating bifurcates in three successive stages. Final prepared pellet samples are tabulated in Table 3.

➢ Drying of pellets occurredin the temperature range of 27–350 °C, within 80 min, and held at 350 °C for 30 min.
➢ Preheating or intensive drying of pellets happened in the temperature range of 350–1000 °C, within 110 min, and held at 1000 °C for 20 min.
➢ The firing of pellets took place at three different temperatures to make 12 sets of pellets.

1. A-set firing was done from 1000 to 1250 °C, within 80 min, and soaking time was 20 min.
2. B-set firing was done from 1000 to 1300 °C, within 80 min, and soaking time was 20 min.
3. C-set firing was done from 1000 to 1350 °C, within 80 min, and soaking time was 20 min.

After firing, the fired pellets (of 8 mm to 16 mm) were collected from the furnace. The weight of pellets was noted as 1.221 g to 5.576 g, as per the size range of 8 mm to 16 mm, and an average size of 10 mm pellet weights were observed as 2.898 g.

### 2.3. Physical and Metallurgical Examination of Pellets

The heat-hardened pellets have been examined for their physical properties: cold compression strength (C.C.S.), and Apparent Porosity (A.P.). The compressive strength of indurated pellets has been measured using Aimil’s Materials Testing Machine, conforming to the Bureau of Indian Standards (IS 8625-1986) [13]. Apparent Porosity (A.P.) of indurated pellets was measured in a kerosene medium, as per the Bureau of Indian Standards (IS 1528) [14]. The prepared pellets for microstructural analysis were mounted in a cold setting compound. The grinding of sets of the sample was done with sandpaper of 220 μm, 400 μm, 600 μm, 800 μm, 1000 μm, and 1200 μm, and they were polished with a 9 μm diamond suspension solution.

### 2.4. Reduction DEGRADATION Index (R.D.I.) and Reducibility Index (R.I.)

The reducibility index was performed, as per JIS M 8713-2000 (Japanese standard), in the 30% CO and 70% N_2_ gas environment, for three hours, at 900 °C. R.D.I. of selected pellets was tested as per JISM8720-2001 (Japanese standard). The average value of each property was taken for analysis [15,16].

### 2.5. Field Emission Scanning Electron Microscopy (F.E.S.E.M.) and Energy Dispersive Spectroscopy (EDS) Analysis

Field Emission Scanning Electron Microscopy (FE-SEM) was conducted for prepared sample pellets with Nova Nano SEM 450 (F.E.I. Company). During Imaging, the chamber pressure was maintained up to −200 Pa and emission current was 99 μA. Image analysis is used to elaborate the information related to phases in the microstructure [17]. EDS was done with X Flash 6130 (Bruker Company). The image analysis tool (ImageJ) was used to measure the area fraction and phase density of different phases in the microstructure [9].

### 2.6. X-ray Diffraction (XRD)

XRD analysis was carried out by Smart Lab 3 kW (Rigaku)—an Automated X-ray Diffractometer—for the phase determination. Fired pellet powder of (−100 mesh) was employed. Radiation Cu-K/opted and 2θ ranges vary from 0 to π/2 during the observation.

Phases identification was carried out by X’Pert HighScore Plus software, R.A.W. files were taken, and background treatment was initiated. Afterward, peaks were searched with 10 minimum significance. Peaks’ search and match were done based on KN/pellet own elements. As per the feasibility, score patterns were selected, and patterns of peaks were determined.

Through converting patterns into phases, determined peaks were quantified. To ensure the given quantification, (expanded Rietveld) refinement was done. After refinement, a better phase percentage was obtained [18].

### 2.7. Fourier Transformation Infrared Spectrum (FTIR)

The FT-IR spectra were recorded in Perkin Etmer, Model Spectrum 2; the data range was 400 cm^−1^ to 4000 cm^−1^ with diamond crystal. The total number of scans was eight, at a pressure 3 to 4 tons, with a KBr binder on the pellet die that is 12 mm in size. FTIR technique assists to observe the iron ore pellet’s behavior in the context of ore fineness and thermal parameters. This analysis shows how the elements are chemically bonded and transformed under a thermal environment, which directly governs the physical and metallurgical properties of pellets by hydroxyl triplet bonding, single bonding, alumino-silicate, loose water, etc. [10].

## 3. Result and Discussion

### 3.1. Green Pellets Characterization

**Figure 2:**
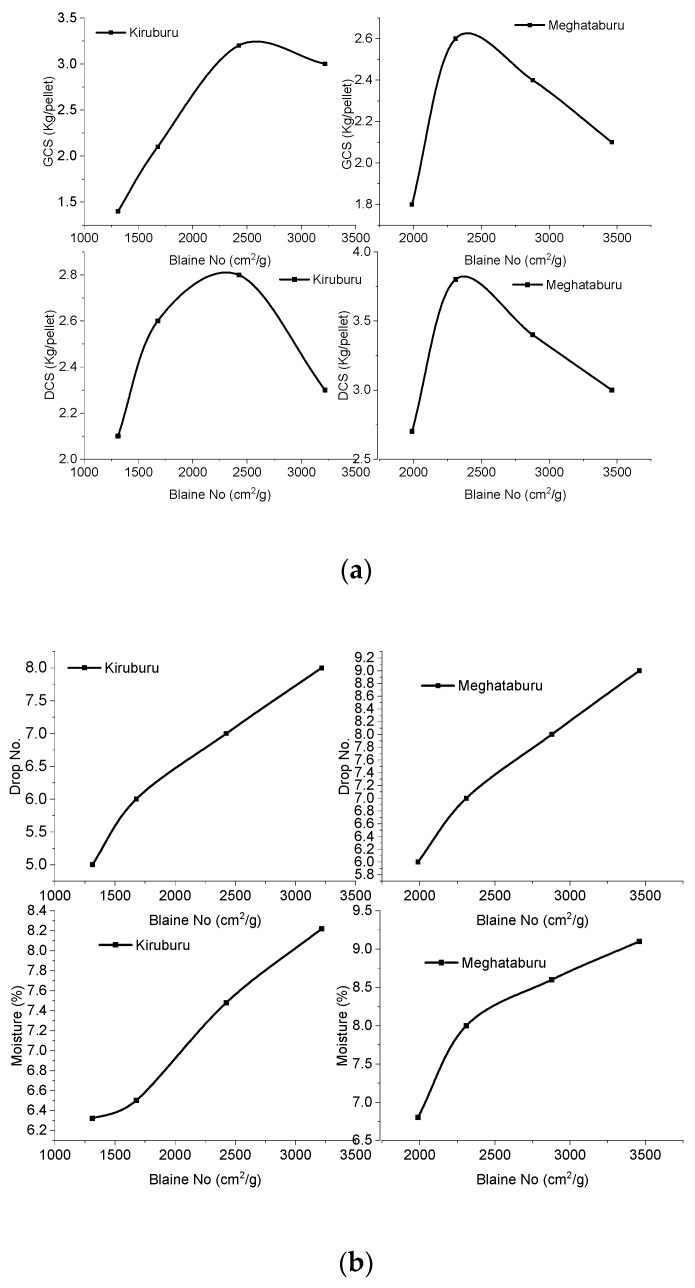
Effect of Blaine fineness (iron ore No.1 and No.2) on (**a**) G.C.S. and D.C.S. (**b**) drop no. and moisture.

Green pellets are taken for their prerequisite properties examination. Green Compressive Strength (G.C.S.), moisture content, Green Drop Strength Number (G.D.S.N.), and Dry Compressive Strength (D.C.S.) were tested, and the results are plotted, as shown, in Figure 2.

G.C.S.: Maximum G.C.S. has been observed at 3.2 kg/pellet for Iron Ore No.1—2425 cm^2^/g pellets and 2.6 kg/pellet for Iron ore No.2—2311 cm^2^/g pellets. Both types of pellets contain <65% of the finer size of −45 µm grains in the pellets. Pellets (Iron Ore No.1—3218 cm^2^/g and Iron ore No.2—2879–3461 cm^2^/g) are having >65% of finer particle proportion, reflecting lower G.C.S. due to the increasing effect of molecular forces resisting the relative mobilization of grains, or it may be due to improper particle arrangement. Finer particles carry moisture in saturation, and this extra moisture may be soaked by dry fines that will be added during pelletizing. This time requires mass transfer through capillaries to get proper green strength [19].

D.C.S.: As the graph reveals that D.C.S. of Iron Ore No.1 pellets increases from 2.1–2.8 kg/pellet, corresponding to 1312–2425 cm^2^/g, Blaine, afterward, decreases to 2 kg/pellet. While iron ore No.2 pellets show growth in D.C.S., from 1989–2311 cm^2^/g, afterward, it reduces from 2.4 to 2.1 kg/pellet. For certain fineness, pellet deterioration occurs, due to the nature of packing. The packing of grains with a gel formation is satisfactory up to an optimum degree of fineness. Afterward, it deteriorates the structure.

Drop no strength (D.N.S.) of pellets shows the plasticity by which pellets can sustain an impact. D.N.S. is increased with fineness, as in Figure 2 for Iron Ore No.1 and Iron Ore No.2, respectively. This is because cohesive capillary forces in the pellet pore increase with finer contents of concentrate and assist in plasticity as well.

Moisture: Moisture continuously increases for both types of ore’s pellets with increasing fineness of ores. This imparts high plasticity, and it also contrutes to the G.C.S./D.C.S. of pellets. This is observed that, up to a certain percentage of moisture, exhibit property well. After a certain percentage of moisture, the degrading of pelletsis initiated. In the present study, an optimumvalue of 7.4% and 8% of the moisture was found for Iron Ore No.1 and 2 pellets, respectively. The lower moisture than this value results in air inclusion and the loss of capillary effects. That results in a lowering of G.C.S. Above this level, extra water covering the surface of pellets nullifies the capillaries’ forces that are reducing G.C.S. Even in dry pellets, a lower level of moisture affects the bonding of the particles and reduces dry strength. Above the optimum level, pellets are left more porous and weakened [10].

### 3.2. Fired Pellets Characterization

#### 3.2.1. Physical Properties (Cold Compression Strength (C.C.S.) and Apparent Porosity (A.P.)) Examination

**Figure 3:**
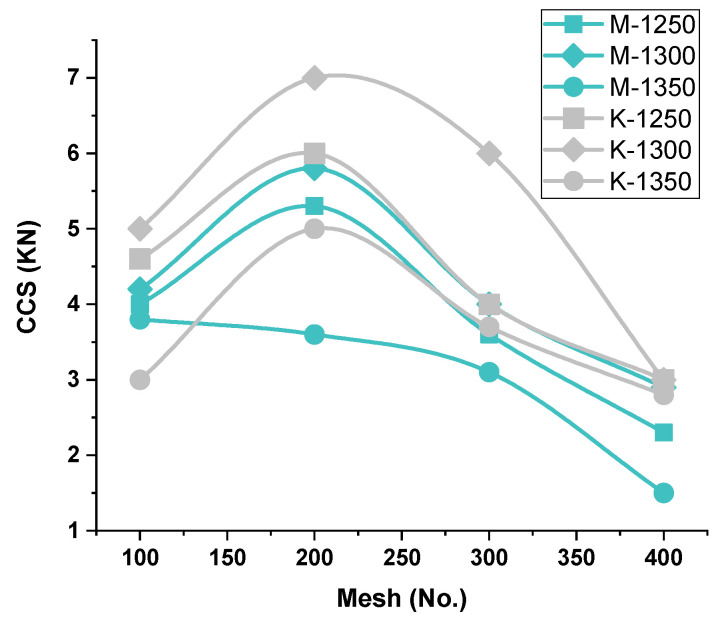
Effect of (Mesh no.) fineness on C.C.S.(KN/pellet)of iron ore No.1/No.2 pellets.

The firing of pellets at 1350 °C provides enough strength (C.C.S.), as shown in Figure 3 C.C.S. of these pellets is a little lower, in value, than the pellets fired at 1250 °C, of both Iron Ore No.1 and 2 pellets of respective Blaine fineness (1312–1678–2425–3218 cm^2^/g of Iron Ore No.1 and 1989–2311–2879–3461 cm^2^/g of Iron Ore No.2). Pellets’ C.C.S. values are in the acceptable range well, which is above the 2.5 KN/pellet. Since both of the mines’ ores are rich in hematite, they require high heat energy for the sintering mechanism during heating. Hematite grains remain less active, up to 1200 °C, resulting in negligible elongation in hematite grains and elimination of defects in the lattice. Up to 1250 °C, initial bridging, crystal growth, and recrystallization commence from the surface shell to the core of pellets [20]. A further mechanism of sintering and oxide bridging succeeded in the presence of coke. On combustion at low temperature, by releasing CO/CO_2_, hematite reduction to magnetite occurs [21].

##### C.C.S.-C.C.S.-vs.-Firing Temperature—Iron Ore No.1 and No.2 Pellets

Since iron ore No.1 and 2 pellets both are hematite ore, hematite begins to react above 1280 °C, while initial crystal formation promotes the strength. Calcium silicate starts to melt and form liquid silicate of calcium ferrite (SCF) at 1250 °C. Al_2_O_3_ prone area SCF reacts with and forms SCFA, which is stable at 1300 °C, and liquid phase calcium silicate and iron silicate slag phase are remaining. Firing at 1300 °C initiates the crystal bridging and recrystallization. Secondary hematite starts to sinter by grain growth in the presence of the slag phase, which enhances the process due to ion migration. The hematite reduction phenomenon produces secondary magnetite (secondary hematite), which contributes to forming slag/silicate melt to promote diffusion bonding [22,23,24]. Hence, the strength of pellets is enhanced. Owing to this behavior, pellets roasted at 1300 °C exhibit a higher C.C.S. value, as compared to pellets fired at other firing temperatures of both Iron Ore No.1 and 2 pellets of respective Blaine fineness (1312–1678–2425–3218 cm^2^/g of Iron Ore No.1 and 2311–2879–3461 cm^2^/g of Iron Ore No.2), as shown in Figure 3.

The lowest C.C.S. is obtained at firing temperature 1350 °C, for both Iron Ore No.1 and 2 pellets, at their respective Blaine fineness (1312–1678–2425–3218 cm^2^/g of Iron Ore No.1 and 2311–2879–3461 cm^2^/g of Iron Ore No.2). Due to increased temperature, high slag/silicate melt formation weakened the pellet structure. Beyond 1350 °C, the temperature of firing hematite dissociation into magnetite and oxygen weakened the strength. The dissociation temperature is decreased with impurities, such as SiO_2_, CaO, and MgO, dissociation of hematite occurs earlier [22]. While firing above 1200 °C, unreacted magnetite core and impervious hematite shell (oxidized magnetite) are developed, which may stop further oxidation of magnetite inside the core, creating a duplex structure, resulting in weakened pellets structure [25].

##### C.C.S.-vs.-Blaine fineness—Iron Ore No.1/No.2 Pellets

In the lower Blaine array (1312/1678 cm^2^/g), Iron Ore No.1 pellets were fired at 1250 °C/1300 °C, showing higher C.C.S. than the Iron Ore No.2 pellets (Blaine 1989/2311 cm^2^/g) due to the Ostwald ripening effect. In which finer particles, present in sufficient proportion, get consolidated faster and provide more strength to the pellets, as shown in size analysis Table 1 and Table 2. Pellet M1C shows higher strength at a firing temperature of 1350 °C. For higher Blaine, the Ostwald effect does not hold satisfactory, conversely increasing fineness. Decreasing strength. Particle size analysis shows Iron Ore No.1 has 46% of 44 microns contributing to better sintering during firing. As compared to Iron Ore No.2 has a 53% of 44-micron excessive fineness makes the grains react more and, instead of transforming solid solution, liquid melt deteriorates the structure. As it can be noted from particle size analysis, more fineness in either iron ore always does not support the strength an optimal value makes the pellets strong [26,27].

##### C.C.S.-vs.-Ore’s Chemistry—Iron Ore No.1/No.2 Pellets

The chemical composition of iron ore fines behaves differently as per the thermal treatment process of pelletization. That is the key role in forming melt phases and sintering grains. Hence, the adversity effect of constituents (SiO_2_ and Al_2_O_3_) is countable to access the economy of iron-steel production. A high composition of alumina in the ore is considered detrimental in pellet production, due to low reactivity and high viscosity of alumina. Therefore, grains are sintered without effectiveness [28]. Siliceous nature creates problems in heat interaction of particles eventually energy consumption increases for getting strength [29].

Iron Ore No.2 ore has gangue content compared to Iron ore No.1 mines as a high Al_2_O_3_/SiO_3_ ratio influences the sintering of iron oxide grains, which results in the viscous melt. That enhances pore size irregularity and affects the pellet sintering [30]. Due to the low reactivity nature of alumina, high viscous melt is expected to be detrimental to the grain-matrix [28]. At high temperatures, slag phases react with alumina, forming SFCA (silico-ferrite of calcium and aluminum), which isa sintering phase with glass and poor sinter matrix strength [31]. Silica blocked the porosity and prohibited the gas flow during the hematite reduction to magnetite, affecting the formation of ý-hematite [29]. High L.O.I. develops fine cracks, during volatile content removal, during the initial phase of drying lower the C.C.S. of pellets. The above points of consideration reveal Iron Ore No.2 pellet’s lower C.C.S., at their respective Blaine array, concerning Iron Ore No.1.

##### AP vs. Firing Temperature—Iron Ore No.1/Iron Ore No.2

**Figure 4:**
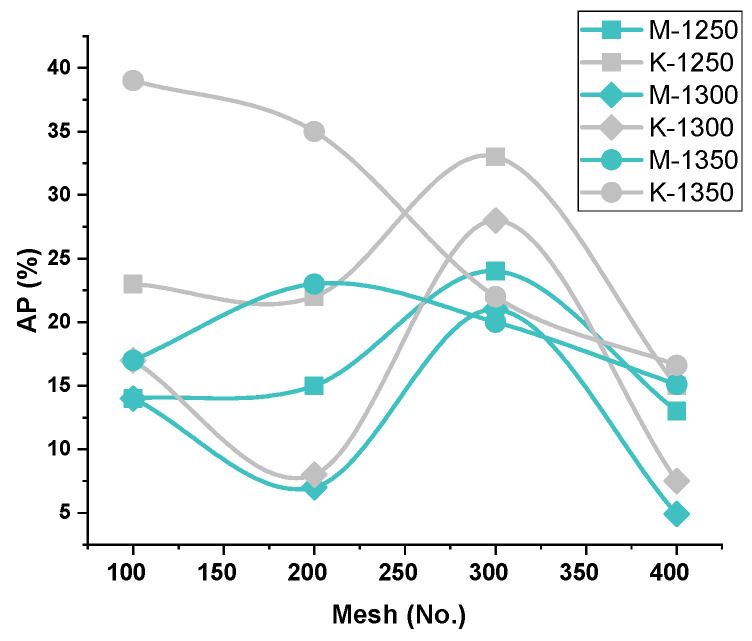
Effect of (Mesh no.) fineness on A.P. of iron ore No.1/No.2 pellets.

**Figure 5:**
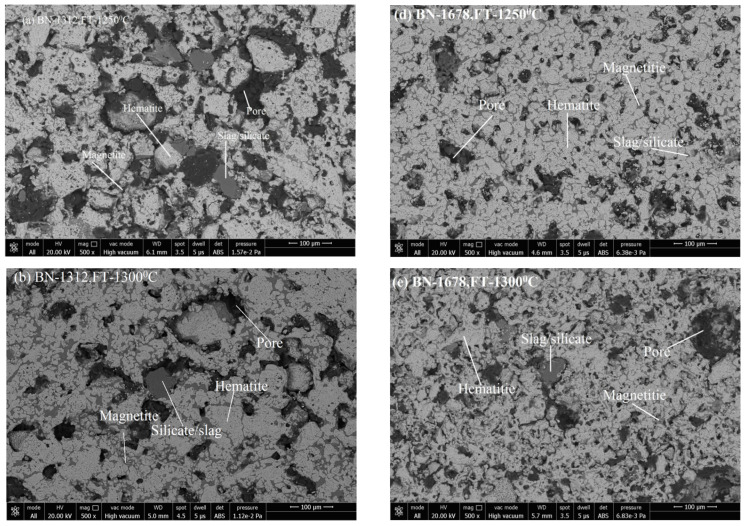

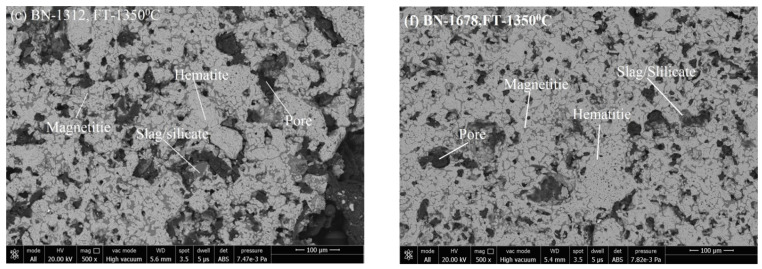
SEM Microstructure of (**a**) K1A, (**b**) K1B, (**c**) K1C, (**d**) K2A, (**e**) K2B, and (**f**) K2C pellets.

**Figure 6:**
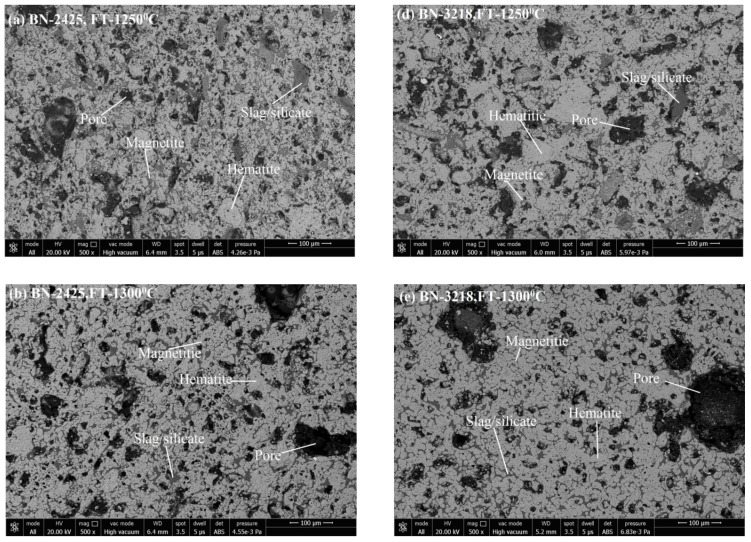

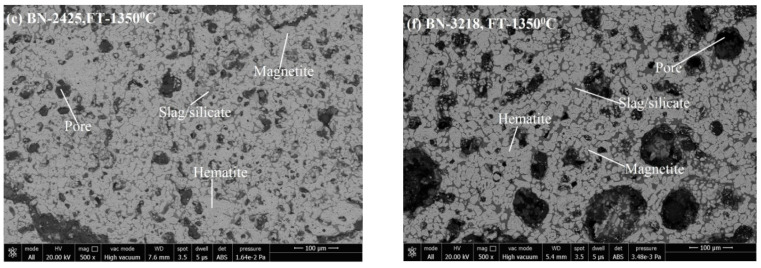
SEM Microstructure of (**a**) K3A, (**b**) K3B, (**c**) K3C, (**d**) K4A, (**e**) K4B, and (**f**) K4C pellets.

**Figure 7:**
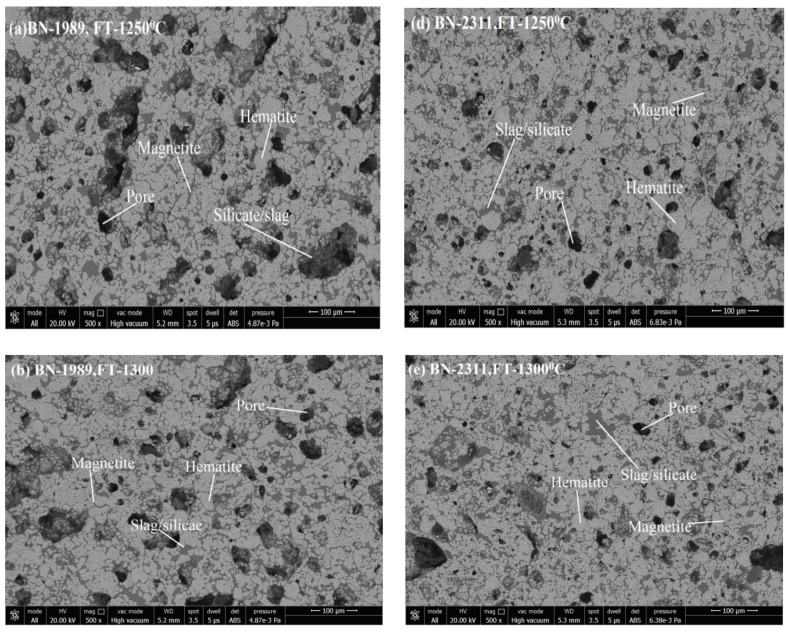

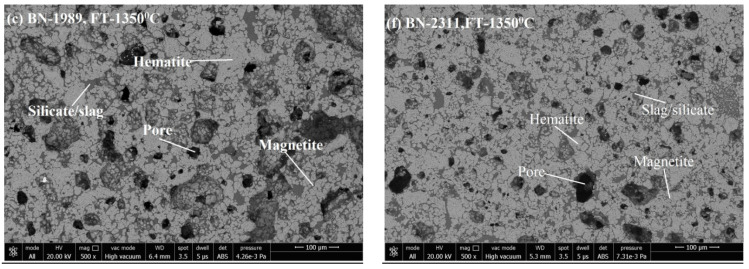
SEM Microstructure of (**a**) M1A, (**b**) M1B, (**c**) M1C, (**d**) M2A, (**e**) M2B, and (**f**) M2C pellets microstructure.

**Figure 8:**
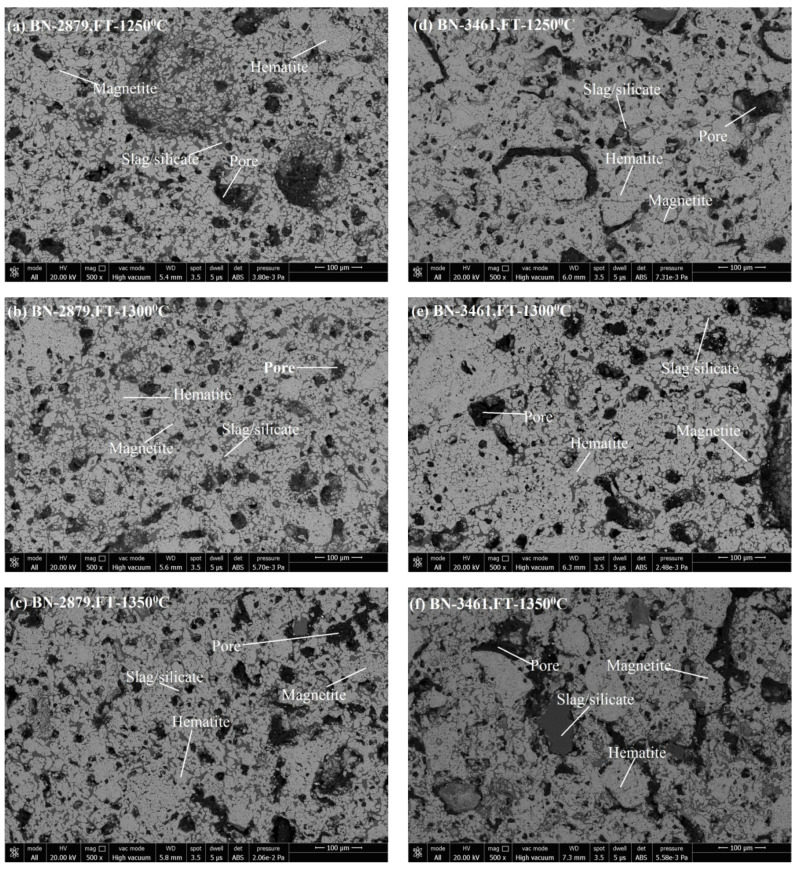
SEM Microstructure of (**a**) M3A, (**b**) M3B, (**c**) M3C, (**d**) M4A, (**e**) M4B, and (**f**) M4C pellets.

Apparent porosity (A.P.) varies conversely to the pellet’s compressive strength, according to the firing temperatures (1250 °C/1300 °C), at the respective Blaine fineness, shown in Figure 4. Besides, the porosity of the pellets, fired at 1350 °C, behave differently due to over-sintering, pore-density, or pore size irregularities. This can be seen in the grain-density analysis of SEM microstructure images in Figure 5, Figure 6, Figure 7 and Figure 8. A duplex structure phenomenon and crystalline transformation generate stresses that cause micropores at a temperature above 1300 °C. The porosity of Iron Ore No.2 pellets falls on the lower side, due to the finer particle proportion of grains, contributing to the finer matrix of pore and iron oxide in the silicates melt phase [32]. The porosity of pellets is caused by various metallurgical processes. At 1250 °C, the initial oxidation and sintering of the silicate matrix, with pores and iron oxides, provide the porosity. CaO additive usage at a different temperature to form calcium ferrite and other ingredients of solid solution and space vacant behind makes pores. When CaO is exhausted, small and uniform grains are closely arranged with fine boundaries, and few porous examples have appeared [9].

##### A.P. vs. Blaine Fineness–Iron Ore No.1/Iron Ore No.2

In the context of Blaine fineness, Iron Ore No.2 pellets show less porosity due to the finer proportion of grains in concentrates. Blaine 1989, 2311, 2879, and 3461 cm^2^/g contain smaller grains than 44 microns in the proportion 48.2, 53.2, 65, and 78 weight %, respectively. This grain proportion of finer particles affects the porosity of pellets. Hence, Iron Ore No.2 pellets possess lower A.P. than Iron Ore No.1 pellets containing coarser fines in more portions [33].

##### A.P. vs. Chemistry—Iron Ore No.1/Iron Ore No.2

**Figure 9:**
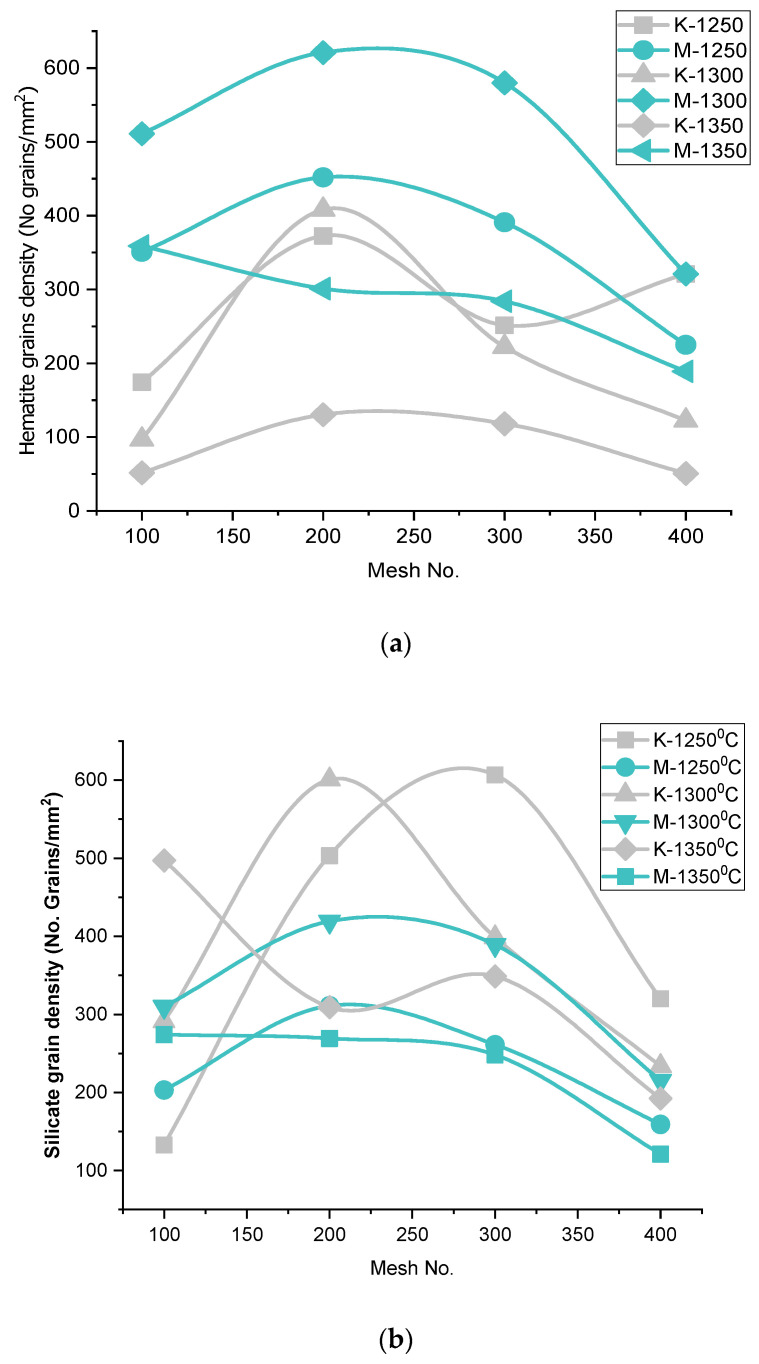
Effect of Mesh no. on Phase Grain density of (**a**) Hematite (**b**) Silicate.

The Iron Ore No.1 pellet shave good enough porosity, as compared to Iron Ore No.2 pellets. A higher melt phase influences pore formation, size irregularities, and unstable phase formation directly influence the porosity of the pellet. Porosity investigation can be done through silicate grains density, and hematite grains density is comparable between both types of pellet origin ores shown in Figure 9. Higher alumina content forms the viscous melt, which deteriorates the matrix. Further, at high-temperature, SFCA formation provides a close arrangement of grains, thereby reducing the porosity [31]. Additional silica closes the pores and reduces the porosity of the pellets, decreasing the reducibility of pellets. Silica prevents particle heat interaction and energy requirement increase. Therefore, Iron Ore No.2 has less porosity, due to high-temperature heating activating alumina as a viscous melt and filling the well-connected pores. Slag formation, due to the reaction of alumina and silica with the additive, connects pores or partially fills the pores.

### 3.3. Microstructure Analysis

Microstructure images reveal the phase formation during thermal processing and the physico-chemical reaction. The microstructure of pellets shows, mainly, primary hematite crystal grains, which are bright with a clear triangular and rectangular shape. Crystal is compact and smooth even though no bond has been formed with grains. Secondary hematite is a bit darker and its residual surface grains have tubular massive grains. During preheating, these grains turn into reticular and vein shapes. As compared to primary grains, the angularities of the secondary hematite grains disappear or become unclear during oxidation. The transformation from massive, columnar shaped particles appeared. At the junction of secondary and primary hematite particles, microcrystalline bonding formed. Secondary hematite is more active than the primary one, mainly contributing to strength [3].

Microstructure images reveal the phase formation during thermal processing and the physico-chemical reaction. Formation of various phases of hematite appears as (grey-white), magnetite (grey-white with pinkish), pores (black shade), and silicate/slag (dark grey) in the microstructure. With its application of grain density in each phase, the size of grains and the phase’s area have been analyzed. Figure 5a–c shows the SEM image of pellets K1A, K1B, and K1C; Figure 5d–f show the SEM image of pellets K2A, K2B, and K2C. Figure 6a–c represent the pellets K3A, K3B, and K3C; Figure 6d–f show the pellets K4A, K4B, and K4C. Similarly, Figure 7a–c show pellets M1A, M1B, and M1C; Figure 7d–f show pellets M2A, M2B, and M2C. Figure 8a–c depict the pellets M3A, M3B, and M3C; Figure 8d–f depict the pellets M4A, M4B, and M4C [10].

#### 3.3.1. Phase Grains Density Study

##### Hematite Phase Grain Density (H.P.G.D.)

Hematite grains, under a reductive environment during heating, reduce to magnetite. This phenomenon occurs in the outer shell of pellets. Oxidation of magnetite produces secondary hematite. As Figure 8 shows, the hematite phase grain density (H.P.G.D.) is the highest at a temperature 1300 °C for the Blaine, corresponding to 200 mesh numbers for both types of ores. The high grain density of hematite depicts the high strength of pellets. Although the C.C.S. value is high for the pellet K2B, comparatively, M2B shows less H.P.G.D. due to the high Blaine 2311 cm^2^/g compared to 1678 cm^2^/g in K2B. This is so because finer particles are more active during the sintering process, and due to fineness, the distribution of grains happens more uniformly. Their distribution shows high grain density, but coarser grains with silicates support the strength. As noted, H.P.G.D. is considerably higher, in respect to Blaine fineness of the Iron Ore No.2 pellet than Iron Ore No.1 pellets, corresponding to firing temperature. The strength of pellets is the collaborative contribution of different phase proportions, with grain distribution corresponding to the firing cycle and ingredients of the pellets mixture. Here, the effect of fineness over the distribution of grains, envisaged as a finer one particle, takes part in the reaction first. Iron Ore No.1 and No.2 pellets, beyond 200 mesh H.P.G.D., decrease due to the increment of finer particle proportion. Firing at a temperature 1350 °C lowers the H.P.G.D. for both types of Iron Ore No.1 and No.2 pellets, showing that, at high temperature, high liquid melt supports faster Fe^3+^ diffusion through melting, and assimilation is prevails to decrease H.P.G.D. [34].

##### Silicate/Slag Phase Grain Density (S.P.G.D.)

The firing of flux pellets of hematite ore exhibit the following reactions. In general, heating of pellets is done at 1000 °C; here, silica and calcium start to react. Very small amounts of calcium form the dicalcium ferrite (Ca_2_Fe_2_O_5_) phase on reaction with iron oxides. It happens only in the secondary hematite region. Calcium dissolves only 2% in magnetite concentrate. At 1100 °C, the core of pellets remain unoxidized, and silica, calcium, and iron oxides begin to react. A phase of calcium and silica-rich appears with larger silica grains. At 1150 °C, slag phases appeared more; slag phases were accommodated between the iron oxide grains. Slag phases were observed to be richer in silica than in calcium [35].

Figure 9 shows that S.P.G.D. for Iron Ore No.1 pellets increases, from Blaine 1312–1678 cm^2^/g, at a firing temperature of 1300 °C. Firing at 1250 °C increases the S.P.G.D. for the Blaine 1312–2425 cm^2^/g, but S.P.G.D. decreases from 1312 to 3218 cm^2^/g of Iron Ore No.1 pellets when fired at 1250 °C. A similar variation of S.P.G.D. Iron Ore No.2 pellets firing at different temperatures was observed to increase from 1989–2311 cm^2^/g; afterward, decreases correspond to 3461 cm^2^/g. Siliceous slag serves as the bonding between the oxide grains. At an appropriate firing temperature, strong slag bond forms provide good strength. Iron Ore No.2 pellets show lower S.P.G.D. for corresponding Blaine and firing temperature.

At temperate 1300 °C, S.P.G.D. is higher for 200 mesh of both Iron Ore No.1 and 2 pellets. With increasing fineness, it decreases at respective firing temperatures. Due to finer particles of silica, other gangue content, and the flux agent, the reaction of silica and CaO forms the slag phase’s primary phase. Later on, quartz, fayalite, CaMgSi_2_O_6_, S.F.C.A., C.F. (calcium ferrite), and 2CF (dicalcium ferrite) phases are formed. These silicate phases are very low (8–10%), proportionately, in the oxide and pore matrix [1].

Proper grain boundaries of the silicate phase iron oxides strengthen the pellets. The S.P.G.D. decreases pore density; hence, apparent porosity (A.P.) and bulk density increase in their respective pellets, as shown in Figure 9. Finer particles in pellets have fused boundaries or assimilated phases that reduce S.P.G.D. in the microstructure [36].

##### Pore Phase Grains Density (P.P.G.D.)

**Figure 10:**
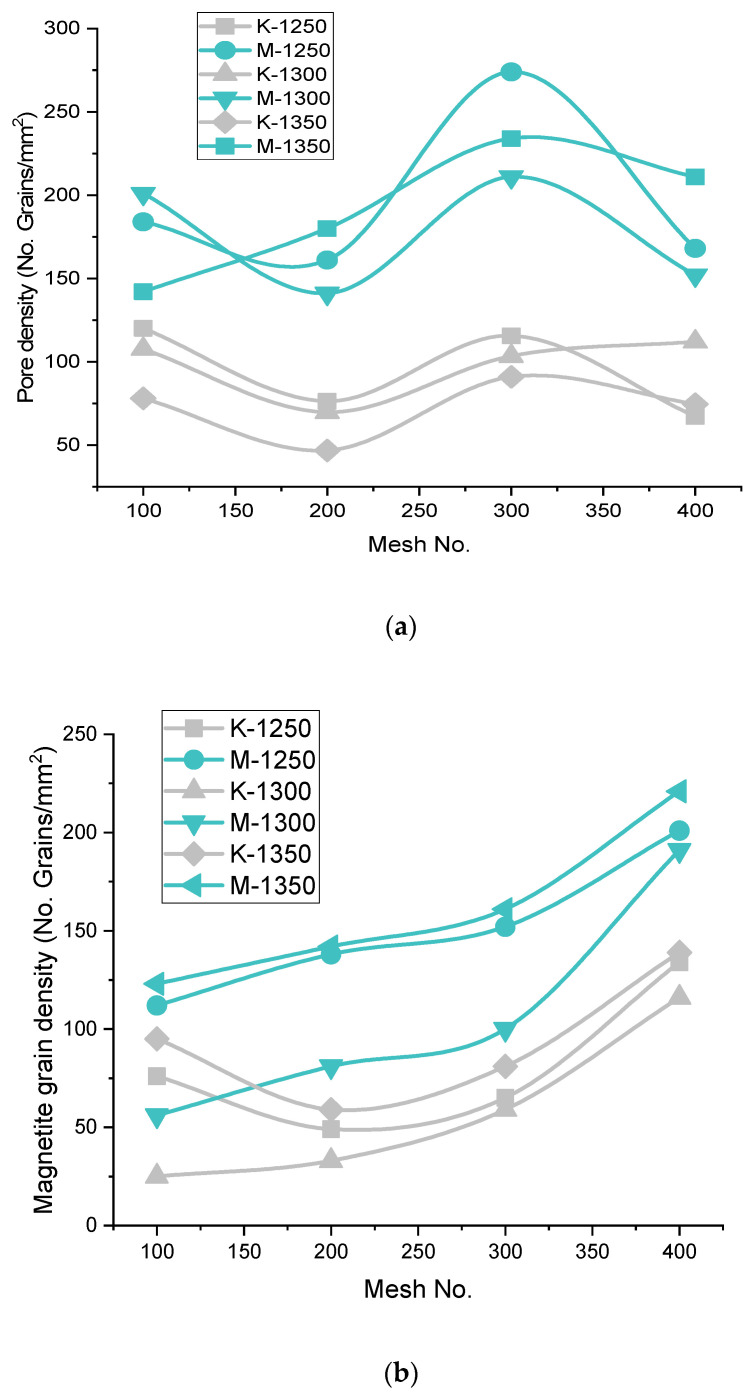
Effect of Mesh no. on Phase Grain density of (**a**) Pores (**b**) Magnetite.

The pore phase is the second major phase, after iron oxide, and the pore phase matrix receives some reinforcement of the silicate phase. Therefore P.P.G.D. decreases as the H.P.G.D. and S.P.G.D. increase in the microstructure. Figure 10 shows that the P.P.G.D. lies on the higher side for Iron Ore No.2 pellets compared to Iron Ore No.1 pellets, due to finer particles. The CaO dissolves faster at the boundaries of smaller particles. After consumption of CaO into its ferrite form, partial or full voids are left over as pores in the matrix [37]. Since high Blaine concentrate pursues finer particles of CaO, smaller pores are formed. Thus, high pore density is achieved compared to coarse fine particles. As the Blaine increases from 1312–1678 cm^2^/g for Iron Ore No.1 pellets and 1989–2311 cm^2^/g for Iron Ore No.2 pellets, P.P.G.D. decreases. The proper sintering of oxides enhances the strength of pellets.

Further increasing Blaine increases (2425 cm^2^/g for Iron Ore No.1 pellet sand 2879 cm^2^/g for Iron Ore No.2 pellets) the P.P.G.D., due to the reactivity of finer oxide with CaO. A smaller size of partial pores appears due to the reaction, as shown in the microstructure of K3ABC and M3ABC pellets [36]. For the finest ore, the Blaine (3218 cm^2^/g for Iron Ore No.1 pellet sand 3461 cm^2^/g for Iron Ore No.2 pellets) corresponds to 400 mesh, assimilation of grains in melt occurs vigorously, phase discrimination becomes difficult even after crystallization, smaller pores become irregular, and bigger pores decrease P.P.G.D. for both Iron Ore No.1 and No.2 pellets [9].

##### Magnetite Phase Grain Density (M.P.G.D.)

M.P.G.D. shows the rate of oxidation of reduced magnetite to secondary hematite, and the grain density of magnetite is observed significantly less as compared to other ingredients. M.P.G.D. increase as the fineness of pellets increases, due to increased resistance of oxidation from the concentric structure that pellets form. Unoxidized magnetite increases with increasing fineness; therefore, M.P.G.D. increases with fineness, which is highest for the pellets fired at 1300 °C; after that, it is highest at 1250 °C, and it is the minimum at 1350 °C for both Iron Ore No.1 and No.2 pellets. M.P.G.D. has more value in the aspect of Iron Ore No.2 due to high oxygen flow resistance. The remaining un-oxidized M.P.G.D. is high as compared to Iron Ore No.1, as shown in Figure 10. The EDS spectra is given in Table 4. 

### 3.4. Energy Dispersive Spectroscopy (EDS) Analysis

**Figure 11:**
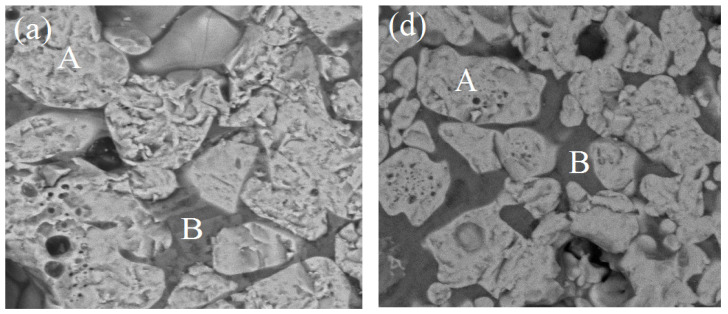

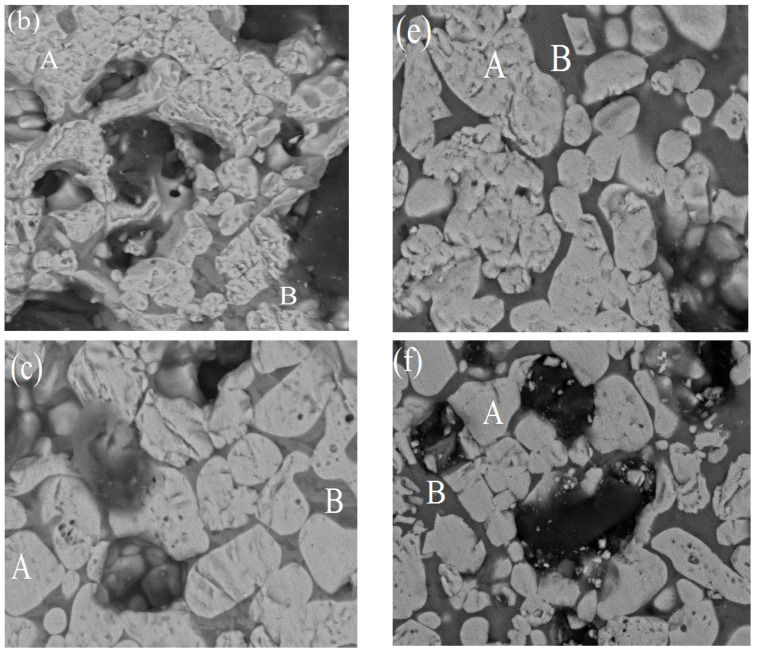
EDS images (**a**–**c**) K2A, K2B, K2C, (**d**–**f**) M2A, M2B, and M2C.

To prove that the phases present in the pellets exhibit physical properties, characterization through microstructure and XRD analysis also chosen as supportive evidence in Figure 11. Few phases were not exhibited in the XRD analysis to support SEM images, EDS analysis provides strong evidence of their presence. As the analysis in Table 4 is shown, the iron oxide phase increases with firing temperature from 1250–1300 °C; afterward, it decreases. For these firing temperatures, ferrite phases are identified when compared to slag bonding firing at 1300 °C. While slag phases predominated when the firing took place at 1350 °C, fineness increases the bonding phase formation at all corresponding firing temperatures, as per the chemistry of the mineral present in the ore.

### 3.5. X-ray Diffraction Study

**Figure 12:**
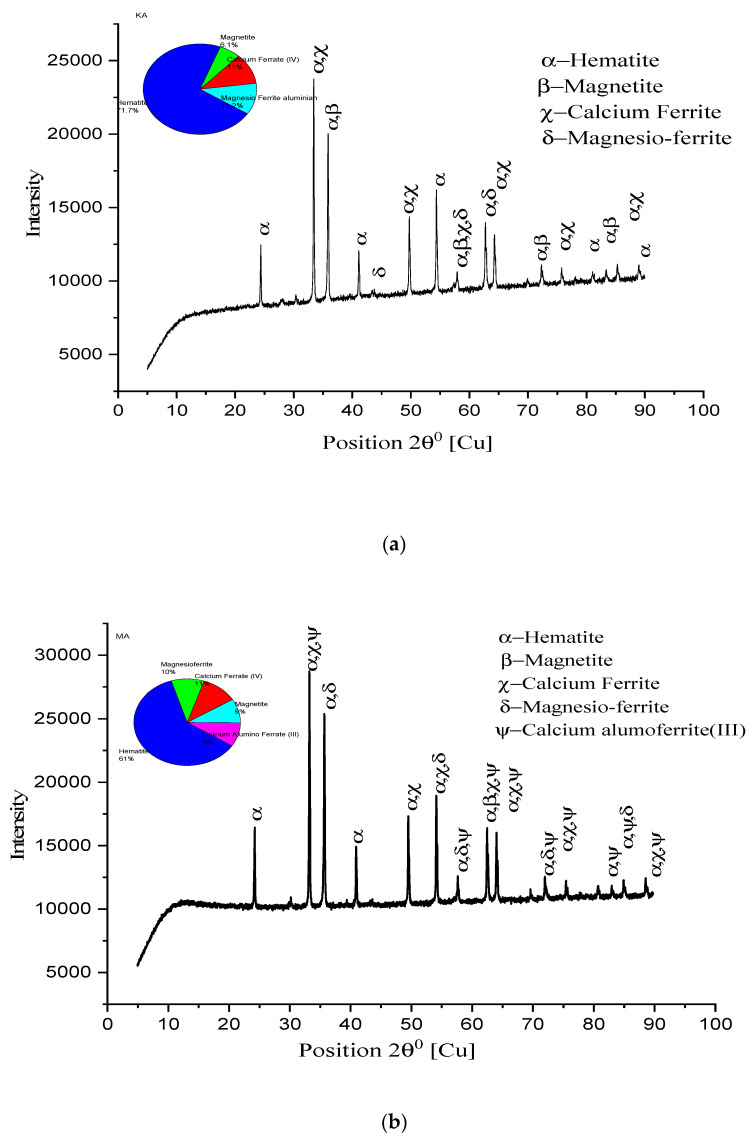
XRD patterns of pellet (**a**) K2A and (**b**) M2A.

**Figure 13:**
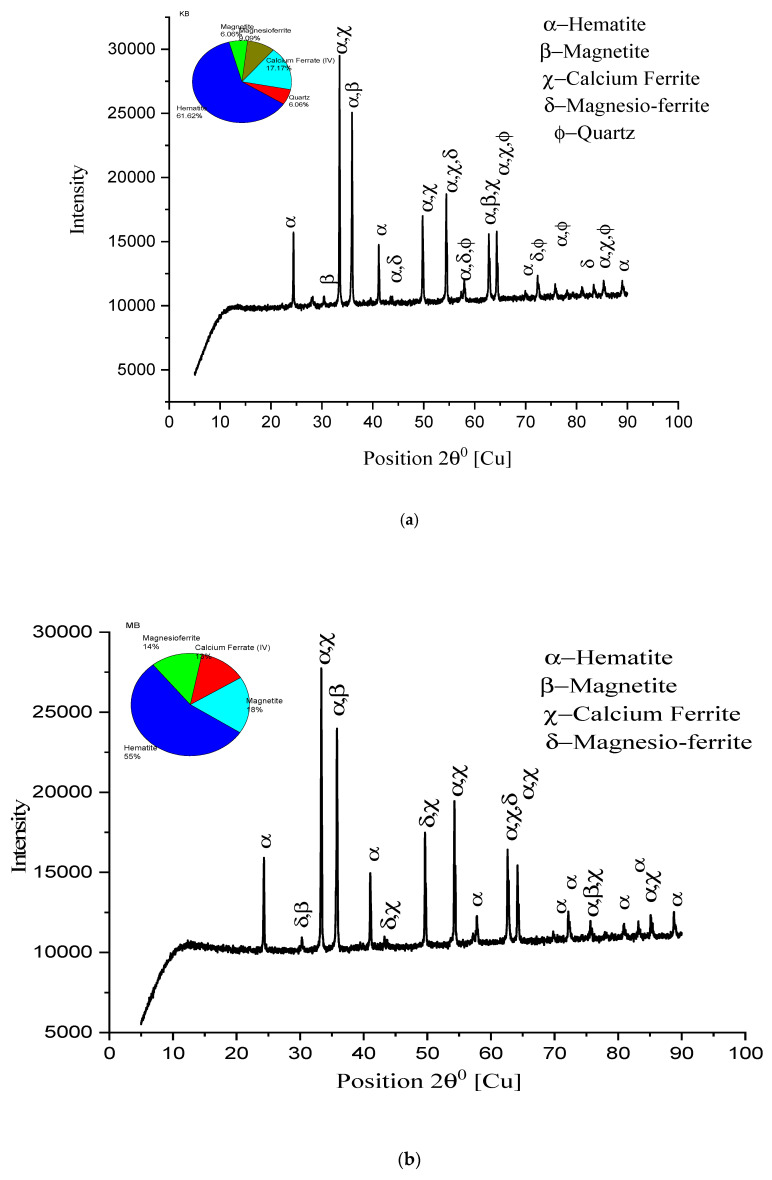
XRD patterns of pellet (**a**) K2B and (**b**) M2B.

An XRD study has been conducted with some remarkable observations to evident the drawn conclusions from SEM analysis. In Figure 12, higher peaks of hematite, along with calcium ferrite and magnesio-ferrite, are shown. Magnetite peaks, with small heights and more in number, are observed. Quartz, along with major constituents, is shown in small quantities). K2A pellets show higher hematite and calcium ferrite peaks, supporting the microstructure images to verify the existing phases in Figure 12a. The percentage of hematite, 71.1%, and ferrite phase, 11%, were recorded. Figure 12b shows that M2A pellet structure’s constituent peaks have hematite and calcium ferrite, and magnesio-ferrite forms the core matrix of pellets to provide strength. Quartz and calcium alumina ferrite, in 9%, are seen in the M2A pellet lowering strength. For the pellet K2B, XRD peaks are shown in Figure 13a. The hematite phase is a major phase (61.1%), while calcium ferrite (17%) and magnesio-ferrite (9%) are secondary phases. Few small traces of magnetite (6%) are shown to show effective oxidation. A high and broader range of peaks is strongly evident in the microstructure image analysis for the pellet’s strength and porosity of pellets. Figure 13b shows the peaks of the M2B pellet’s phases existing in the microstructure. The quantity of major phases reduces, while magnetite (18%) content increases concerning K2B pellets, differing in pellet strength [18].

**Figure 14:**
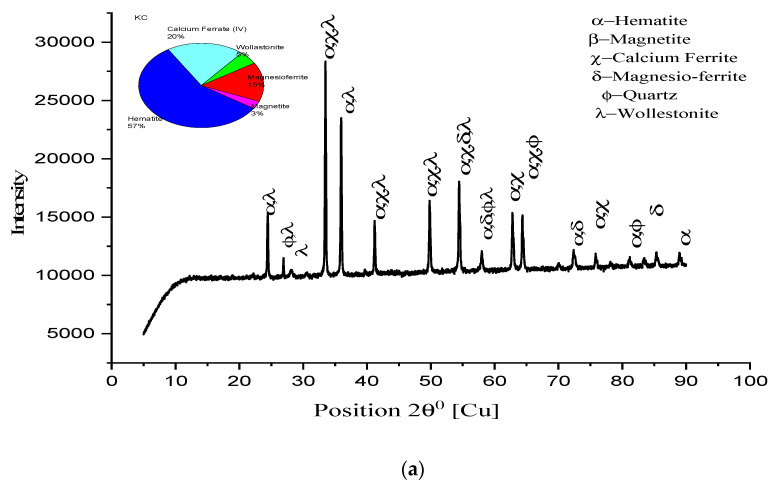

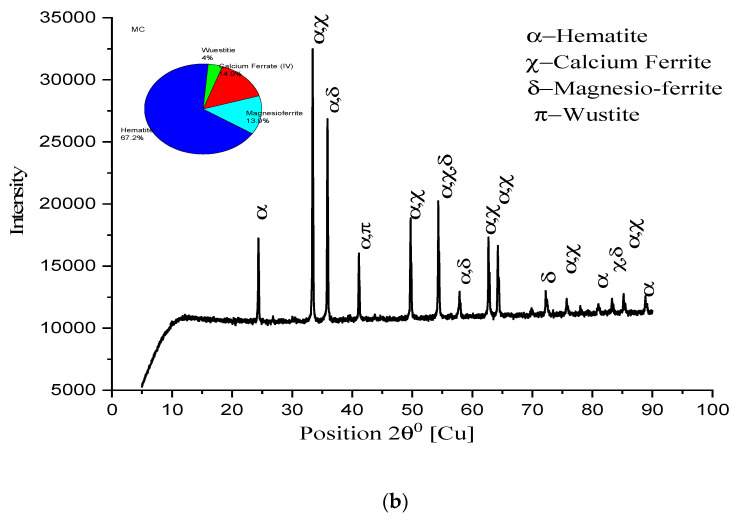
XRD patterns of pellet (**a**) K2C and (**b**) M2C.

In Figure 14, peaks of the hematite (57%), calcium ferrite (20%), and magnesio-ferrite slag phases are seen in both K2C and M2C pellets. Hematite (57%), calcium ferrite (20%), and magnesio-ferrite (15%) are observed for K2C, and hematite (67%), calcium ferrite (14.9%), and magnesio-ferrite (13.9%) are observed for M2C. Two different phases of wollastonite (5%) in K2C and wustite (4%) in M2C were observed. These phases reduce the pellet’s strength and appear through prolonged magnetite reduction to wustite/wollastonite. Moreover, M2C has higher hematite due to fineness, but it shows less strength than K2C.

The above discussion of XRD analysis supports optimizing the firing temperature, as the firing temperature is 1350 °C, and the hematite phase % reduces, either due to the dissociation of magnetite or the reversion of hematite.

Microstructure, as appears in Figure 5d–f and Figure 6d–f of pellets K2A, K2B, K2C, M2A, M2B, and M2C, respectively. Firing at temperature 1300 °C performs proper sintering of grains, and the ferrite phase forms a matrix of hematite grains to support the strength of pellets’ structure and optimum porosity, as well. Although firing at 1250 °C produces pellets of desirable properties, a slightly noticeable change in properties occurs due to the refined microstructure of clearly visible grain boundaries and well-connected oxide phases [38].

Figure 13a,b are the peak representation of pellets K2B and M2B, respectively, as the heights of peaks of the hematite and calcium ferrite phases are greater in numbers. Peak height represents the intensity of the phase available within the microstructure: hematite phase 61.6% of K2B; 55% of M2B and calcium ferrite phase 17% of K2B; 13% of M2B shows the highest strength of pellets. Only magnetite phase 9% of K2B and 14% of M2B lower the M2B strength, which shows that an unavoidable phenomenon occurred. Figure 12a,b and Figure 14a,b peak availability and phase percentage are strongly evident in the microstructure phase grain density analysis to validate the physical properties (C.C.S. and A.P.).

### 3.6. Fourier Transform Infrared Spectroscopy (FTIR)–Analysis

Iron Ore No.1 and No.2 pellets of the highest strength, moderate strength, highest porosity, and least strength were examined under FTIR spectroscopy in the respective chapter. Here comparative analyses concerning the abovementioned context are discussed [39].

#### 3.6.1. Highest C.C.S. Pellets

**Figure 15:**
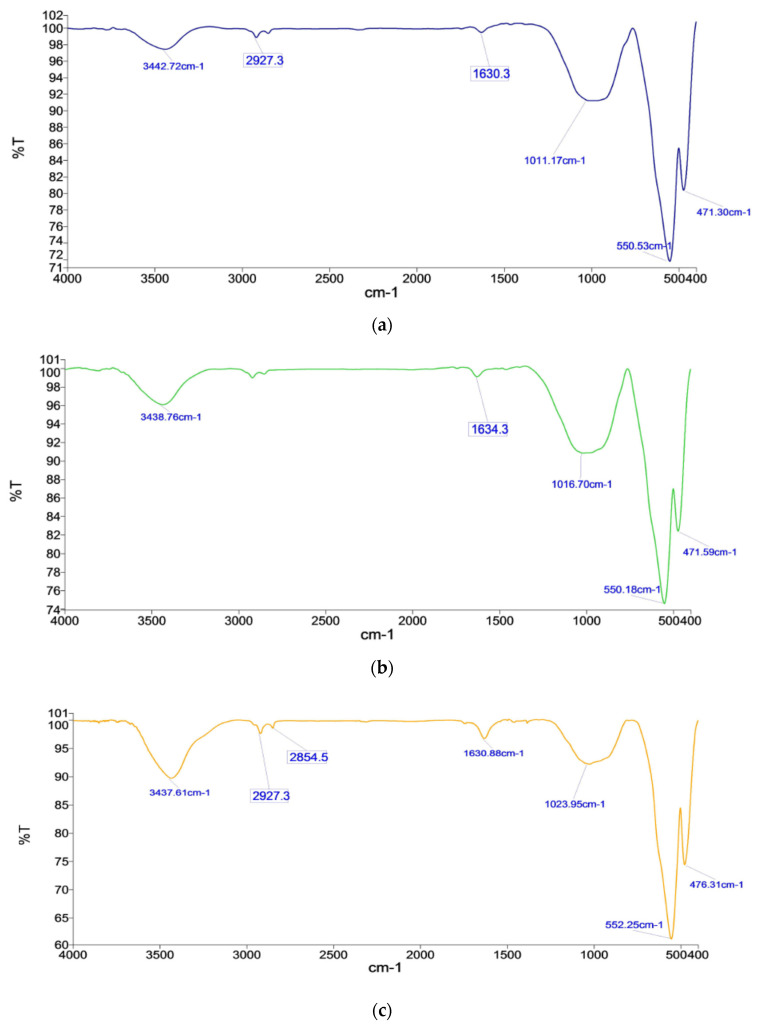

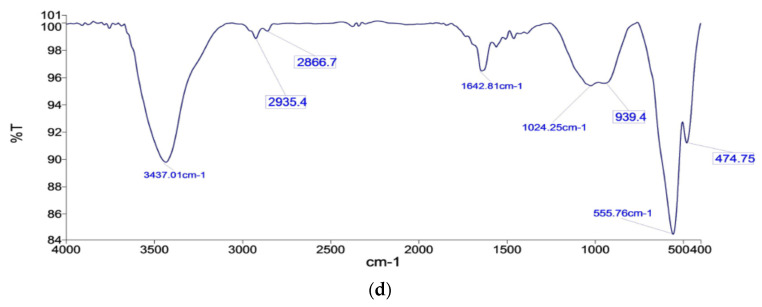
FTIR spectrographs of pellets (**a**) K1A, (**b**) K2B, (**c**) K3A, and (**d**) K4C.

**Figure 16:**
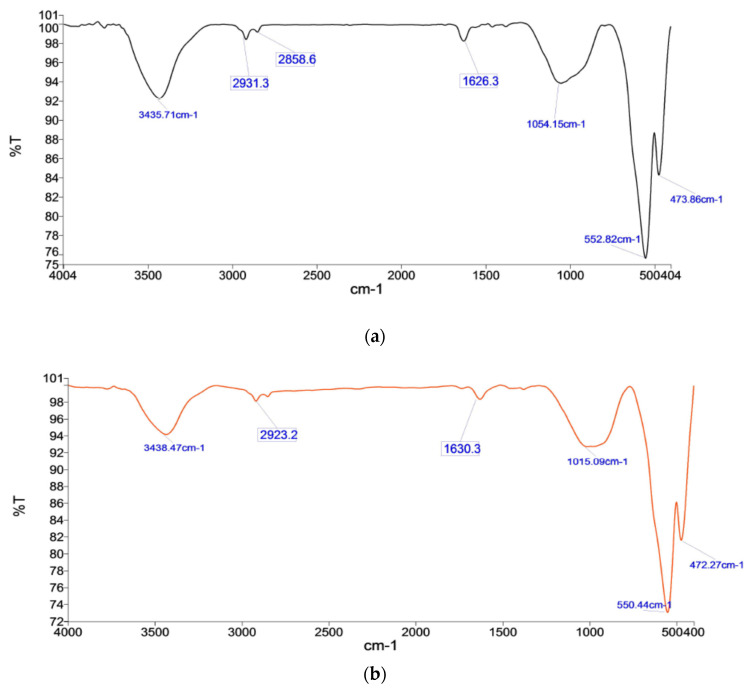

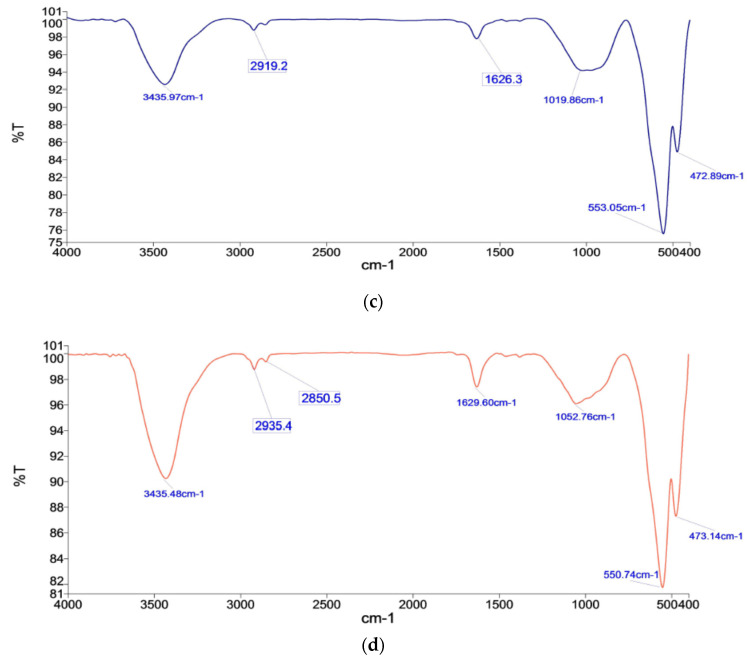
FTIR spectrographs of pellets (**a**) M1A, (**b**) M2B, (**c**) M3A, and (**d**) M4C.

Pellets K2B and M2B have the highest C.C.S. due to significant hematite and silicate peaks with fewer peaks or ignorable peaks of hydroxyl content. Since K2B has higher C.C.S., due to the presence of the hematite compound, of 471.59 cm^−1^ and 550.18 cm^−1^, M2B phase peaks 472.27 cm^−1^ and 550.44 cm^−1^, with significant absorbance, as shown in Figure 15b and Figure 16b. Here, M2B shows a bit more absorbance due to finer grains showing abundance in the material, but K2B has a broader range. Slag/silicate phase bonds provide strength; silicate in K2B has 1016.70 cm^−1^, which is broader and deeper absorbance as compared to the M2B silicate peak (1015.09 cm^−1^). The high L.O.I. hydroxyl group peak’s 3438.47 cm^−1^ 2923.2 cm^−1^, weakened the structure. Pellet strength depends on the distribution of iron oxide phase grains and how they are configured in the silicate/slag phase with the pores matrix. Firing at temperature 1300 °C, physico-chemical reactions turn in the presence of coke, and various products are formed. Crystal transformation occurs swiftly, as evident in FTIR and XRD phase analysis. Iron oxide compound bonding (hematite) spectroscopy (for K2B 471.59 cm^−1^ and 550.18 cm^−1^; for M2B 472.27 cm^−1^ and 550.44 cm^−1^) was observed. Silicate/slag compound bond affiliation, denoted by the peak (for K2B 1016.70 cm^−1^; for M2B 1015.09 cm^−1^) in a broader range, shows dominance in the microstructure.

#### 3.6.2. Significant C.C.S. Pellets

Pellets K1A and M1A show significant C.C.S. after K2B/M2B pellets, in their respective places, and K1A shows good strength compared to M1A. K1A has 471.30 cm^−1^ and 550.53 cm^−1^ great absorbance of 71%, compared to M1A pellets of hematite’s peaks, 473.89 cm^−1^ and 552.82 cm^−1^, which have 75% absorbance, as shown in Figure 15a and Figure 16a. Along with this silicate peaks in K1A, 1011.17 cm^−1^ has high absorbance compare to the M1A silicate peak (1054.15 cm^−1^). K1A containa lower hydroxyl element compared to M1A and shows a bit more, at 3442.72 cm^−1^ and 3435.71 cm^−1^, respectively. Pellets fired at 1250 °C and crystals of iron oxide phase, formed after preheating a sufficient iron oxide compound, were evident, by FTIR spectroscopy, as 471.30 cm^−1^ and 550.53 cm^−1^ for K1A and 473.89 cm^−1^ and 552.82 cm^−1^ for M1A. Since both ores are rich in hematite, oxide bonds were observed up to 1300 °C.

#### 3.6.3. Highest Porosity Pellets

Pellets K3A and M3A have high porosity among other processed pellets, K3A has more porosity than M3A, as hematite phase peaks of 476.31 cm^−1^ and 552.25 cm^−1^ have good absorbance compared to 472.89 cm^−1^ and 553.05 cm^−1^ but less in wavelength. K3A has low absorbance of silicate peaks, and 1023.95 cm^−1^ (compared to 1019.86 cm^−1^ of M3A) shows a porous silicate phase, as shown in Figure 15c and Figure 16c. Apart from this hydroxyl group, loose water with some more peaks of the O-H bond, have been observed as 3437.61 cm^−1^, 2927.3 cm^−1^, 2854.5 cm^−1^, and 1630.88 cm^−1^, while M3A has only 3435.97 cm^−1^, 2919 cm^−1^, 1626.3 cm^−1^.

#### 3.6.4. Minimal C.C.S. Pellets

The pellets K4C iron oxides (hematite) peak 474.75 cm^−1^ and 557.66 cm^−1^ good enough compare to M4C, less absorbance of iron oxides (hematite) peak 473.14 cm^−1^ and 550.74 cm^−1^, as shown in Figure 15d and Figure 16d. Silicate/slag phase peaks, 1042.25 cm^−1^ (for K4C) and 1052.76 cm^−1^ (for M4C) with higher wavelengths, provide a little more strength. The firing temperature reached 1350 °C, and a liquid silicate phase occurring due to over sintering and unstable phases were seen. Such unstable compound phases are captured as a stretched bond group. The hydroxyl group and stretched O-H groups stretched and sharp bands have appeared as the peaks 3437.01 cm^−1^ and 2866.7–2935.4 cm^−1^, respectively, for K4C. These peaks marginally influence the pellet’s structural strength, as compared to M4C pellets (hydroxyl group and stretched O-H groups stretched) peaks 3435.48 cm^−1^ and 2850.5–2935.4 cm^−1^ with less negative influence on the strength of pellets [10].

### 3.7. Reduction Degradation Index (R.D.I.) and Reducibility Index (R.I.) of Pellets

The degradation of pellets tends to generate fines under low-temperature reduction in the blast furnace. This generation of fines occurs due to various reasons, such as hematite to magnetite (hexagonal to cubic) transformation, leading to volumetric expansion. Expansion of pellet structure initiates fine crack due to stress development. For the pellets in blast furnace application, RDI in the fraction of −3.15 mm is considered, and around 20% is tolerated in blast furnace [40]. FeO percentage in the pellets influences the R.D.I., as the content is increasing with firing, as in this case for Iron Ore No.2 at 1350 °C, wustite is formed and increases the R.D.I. [41]. The formation of the melting slag phase disturbs the structure of the pellet and increases the R.D.I., as the Iron Ore No.2 pellets slag phase is higher than Iron Ore No.1 pellets, corresponding to all firing temperatures in increasing order [40,42]. Iron Ore No.2 has a higher R.D.I. compared to Iron Ore No.1, due to Al_2_O_3_ content being high, as a solute in hematite responds to high R.D.I. [30]. Alumina in hematite develops a distorted weak structure at a low-temperature reduction [43,44].

The type and distribution of phases within the microstructure also reflect physico-chemical properties. Microstructure analysis and XRD phase analysis validate the R.D.I. and R.I. variation in the selected pellets study. High hematite grain density (H.P.G.D.) shows improper sintering of grains as Iron Ore No.2 ore pellets have the same. Firing at 1350 °C, a high H.P.G.D. shows high R.D.I., as compared to pellets firing 1300 °C/1250 °C has low R.D.I. Pore phase density is also high for Iron Ore No.2, as well as higher at 1350 °C than 1300 °C. The high phase percentage shows high R.D.I. for pellets fired at 1250 °C. Iron Ore No.1 pellets have P.P.G.D. in the order of 1250 °C to 1350 °C, showing the R.D.I. growth in the same order. Silicate grain density is high for Iron Ore No.1 has improved R.D.I. than Iron Ore No.2.

**Figure 17:**
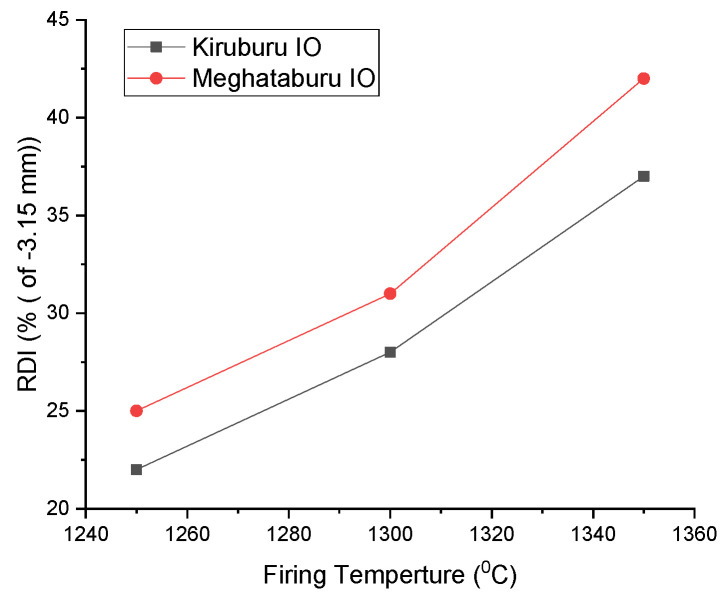
Effect of Firing Temp. on R.D.I.

Firing at 1300 °C produces greater strength than 1250 °C but R.D.I. is increased, as per heating, due to bigger pore formation and distribution of ore, causing high R.D.I., as shown in Figure 17. Uneven, the pellet structure at high-temperature hematite ore with coke decreases the pellets’ sintering; hence, R.D.I. increases.

Reducibility index (R.I.) is the ease with which oxygen is removed. For good R.I., pellets should ensure enough porosity. Iron Ore No.1 pellets have coarser fines compared to Iron Ore No.2 pellets and show good apparent porosity (A.P.) and offer good R.I. As the firing temperature increases provide greater sintering and good bulk density (B.D.), it reduces the porosity; hence, R.I. decreases. Reversion of magnetite in the CaO presence produces magnetite inclusion to fulfil the intertidal between the hematite grains that are replacing oxide bonds, resulting in a weaker structure of pellets [27,45]

**Figure 18:**
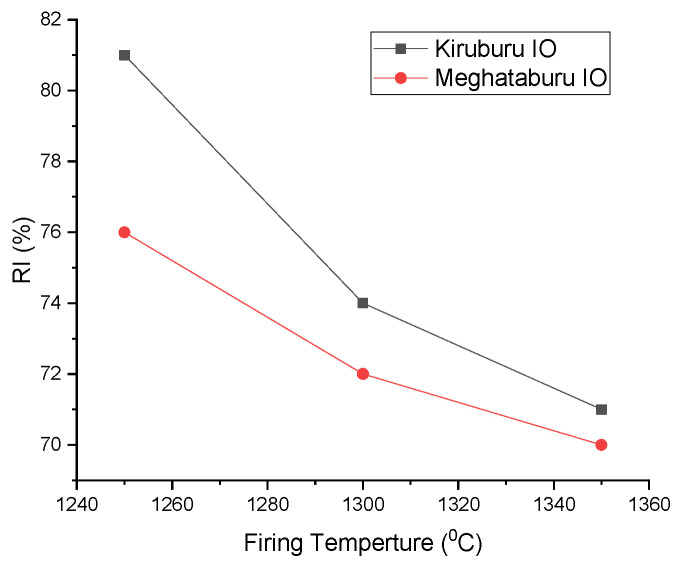
Effect of Firing Temp. on R.I.

The fineness of ore and the bonds between the grains affect the pellet’s reducibility. Porosity of pellets supports the reducibility. Carbon addition accesses the various reactions, such as hematite reduction-produced magnetite conversion, to secondary hematite, which is less reducible than primary hematite because increasing the firing temperature lowers the reducibility. Iron Ore No.2 pellets have more fineness preventing oxygen removal, which decreases the reducibility [21,38]. The pellets fired at 1250 °C reflect the higher reducibility since they have a lower magnetite phase and less silicate/slag bond with a higher pore phase. Pellets fired at 1350 °C have more slag bonding with reduced pore phase, less density, and more magnetite grains, presenting the lower reducibility shown in Figure 18 [4].

### 3.8. Thermal Kinetics Analysis of Heating of Pellets

#### Pellets Heat Treatment Can Be Divided into Three Steps

(a) Drying at 250–400 °C (b) Preheating 900–1200 °C (c) Firing at 1200–1350 °C

To investigate the heating rate effect on the dissociation of the hematite oxide phase of ore. After weighing each time of the different experiments, the iron ore concentrate sample was put in an alumina pan. During heating, approximately 20 mg weight of ore was maintained, and argon gas was being blown at 200 mL/min. The reference sample of alumina powder was taken for different heating rates. In the present work, hematite ore is used and associated with dissociation at high temperatures during firing. This deteriorates the strength and reducibility of pellets. Dissociation of hematite may be, of any type, secondary or naturally present in the pellets. A researcher found hematite stability in the context of particle size and temperature. Dissociation starts at 1264 °C and ends at a temperature of 1392 °C in the air [46]. Although this is a temperature control reaction, magnetite oxidation, having an exothermic nature, makes the process complex. Thus, this is imperative to understand, comprehensively, temperature relevance with dissociation kinetics. A thermal kinetics analysis provides kinetic parameters of the reaction associated with the phenomenon. As per the availability of data and users’ convenience, methods were adopted for analysis. The fitting approach model has advantages of both kinetic and mechanism process constant.

In the present study, Coats and Redfern’s method is used to do a kinetic analysis of non-isothermal Thermo-Gravimetric (TGA) data [47,48]. Higher activation energy denotes strong temperature dependence of reaction. A small increment in the temperature results in faster dissociation of reaction. Three heating rates are recommended for the kinetics study to elaborate the complete course of action.

To compute the activation energy for the thermal decomposition process, leading to the formation of spinel magnetite ferrite, the TGA data has been analyzed using the Coats–Redfern method. According to the Coats–Redfern method, the mathematical relation for a first-order reaction is expressed as

(1) $$\log\left[\frac{-\log\left(1-\alpha \right)}{T^{2}}]=\;\log\;\frac{\mathrm{AR}}{{\beta E}_{a}}[1-\frac{2\mathrm{RT}}{E_{a}}\right]-\frac{E_{a}}{2.303\mathrm{RT}}$$

where α is the fraction of sample decomposed at time t given by

$$\alpha =\frac{W_{o}-W_{t}}{W_{o}-W_{f}}$$

*W_o_* is the initial weight of the sample, *W_t_* represents the weight of the sample at any given temperature, and *W_f_* is sample weight after the completion of the reaction. Βis the linear heating rate, T is the absolute temperature, E_a_ is the activation energy, A is the frequency factor, and R is the gas constant (8.314). By using Equation (1), a graph is drawn between $\log\left[\frac{-\log\left(1-\alpha \right)}{T^{2}}\right]$ and $\frac{1000}{T}$.

Finally, the slope of this linear graph can be written to calculate the activation energy of the reaction. For the optimality of reaction kinetics, the conversion value of 0.5 has been taken.

For the kinetics analysis of ore samples, Iron Ore No.1 and No.2 are taken off close to Blaine fineness. TGA data has been analyzed for the mass loss percentage with temperature.

**Figure 19:**
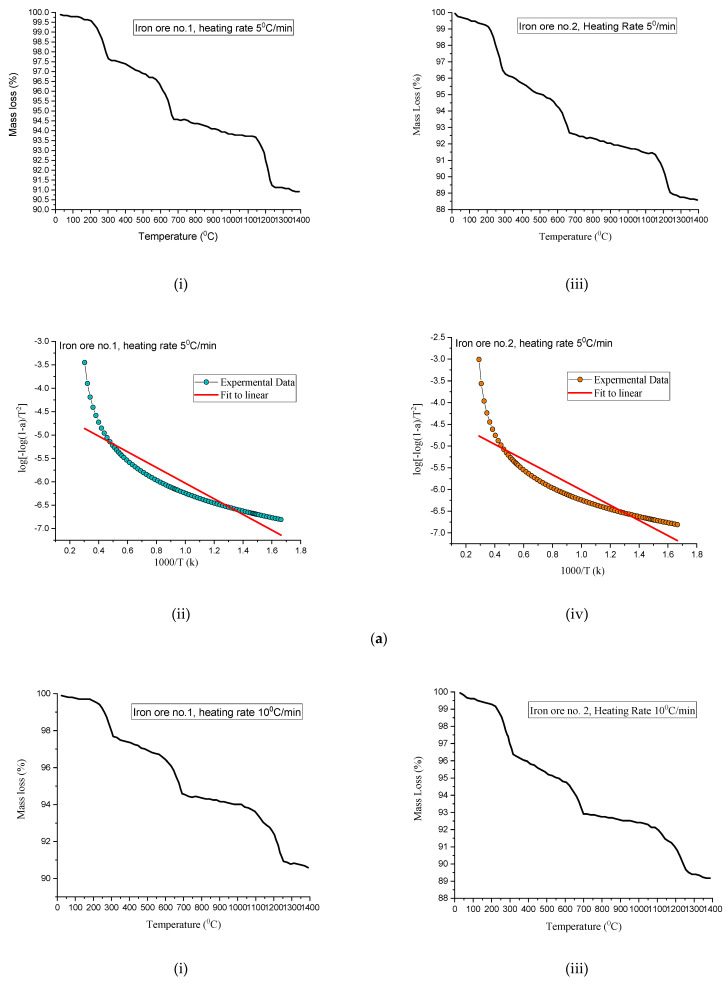

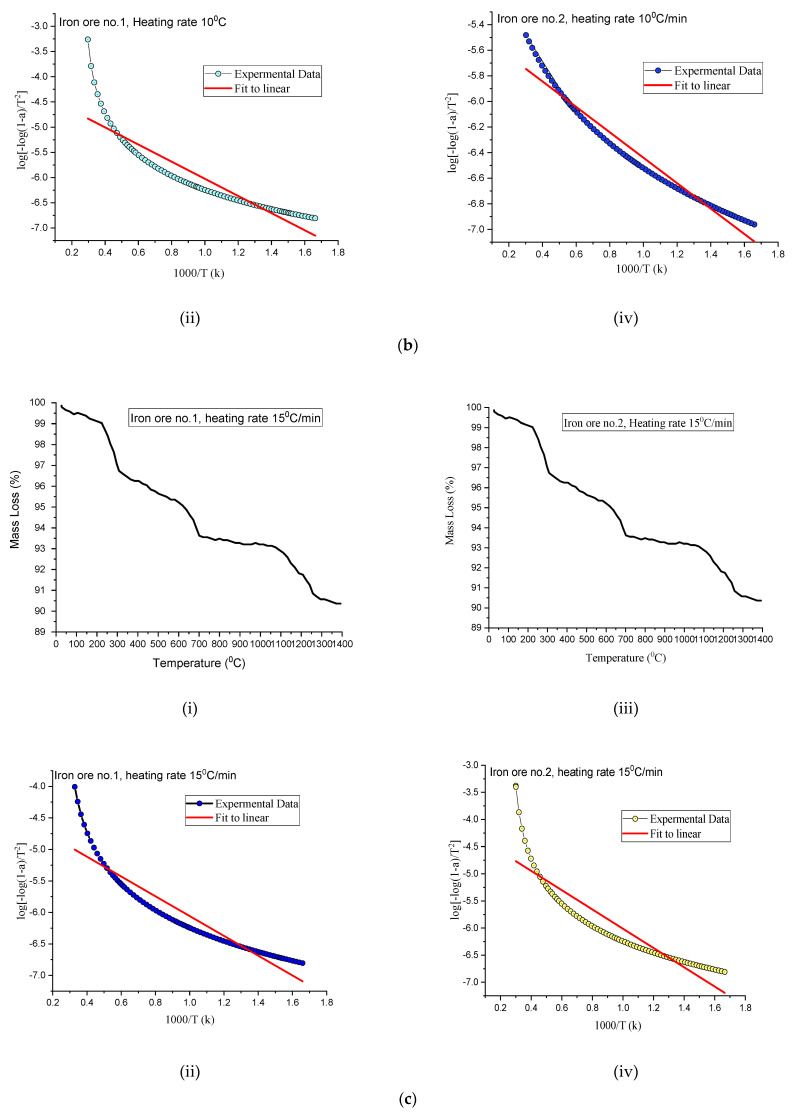
(**a**) Mass percentage with temperature of (i) Iron Ore No.1 and (iii) Iron Ore No.2; Experimental data fitting the curve of (ii) Iron Ore No.1 (iv) Iron Ore No.2 at a heating rate of 5 °C/min. (**b**) Mass percentage with temperature of (i) Iron Ore No.1 and (iii) Iron Ore No.2; Experimental data fitting the curve of (ii) Iron Ore No.1 and (iv) Iron Ore No.2 at a heating rate of 10 °C/min. (**c**) Mass percentage with temperature of (i) Iron Ore No.1 and (iii) Iron Ore No.2; Experimental data fitting the curve of (ii) Iron Ore No.1 and (iv) Iron Ore No.2 at a heating rate of 15 °C/min.

Figure 19 shows (i) mass percentage versus temperature graph, (ii) linear graph for Iron Ore No.1, (iii) mass percentage versus temperature graph, and (iv) linear graph for Iron Ore No.2, when heating rate 5 °C/min was considered. Similarly, Figure 19b,c show that the same mentioned parameters correspond to heating rate of 10 °C/min and 15 °C/min, respectively.

As shown in Figure 19a–c, experimental data (TGA) mass loss percentages with increasing temperatures were plotted, corresponding to heating rates 5 °C/min, 10 °C/min, 15 °C/min, respectively. Conversion (α) can be chosen concerning any temperature point on a mass percentage graph. For the sake of optimality, 0.5 conversions were chosen and correspond to this $\log\left[\frac{-\log\left(1-\alpha \right)}{T^{2}}\right]\;\mathrm{vs}.\;\frac{1000}{T}$ was drawn. After the linear fitting, the data slop of line is considered, as listed, in a and b in Table 5. The activation energy has been calculated as;

$$-\frac{E_{a}}{2.303\;R}=slope$$

**Figure 20:**
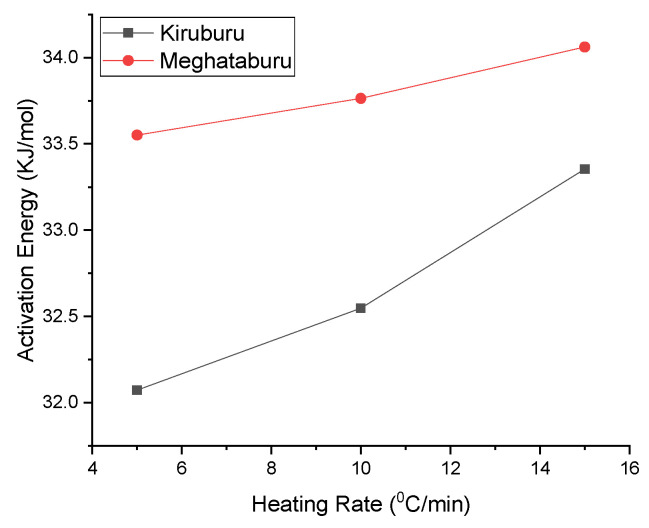
Effect of the heating rate on activation energy.

Hematite dissociation is the undesirable reaction during induration for this non-isothermal kinetic model has taken for analysis. A small temperature change governs the hematite dissociation, along with the fineness of ore. TGA was done to get data for conducting kinetic analysis and concluded that the dissociation reaction rate is temperature-dependent: the higher the activation energy value, the higher the temperature dependence. Figure 20 shows that activation energy increases with the heating rate, influencing the hematite dissociation to become more severe with a high heating rate. As compared to Iron Ore No.2, the sample has high activation energy, corresponding to the same heating rate of Iron Ore No.1, and owing to the fineness of ore particles being high for Iron Ore No.2 compared to Iron Ore No.1.

## 4. Conclusions

The following salient points have been concluded.

➢ Pellets roasted at 1300 °C exhibit a higher C.C.S. value than the pellets fired at other firing temperatures, of both Iron Ore No.1 and No.2 pellets, of respective Blaine fineness. In the lower Blaine array (1312/1678 cm^2^/g), Iron Ore No.1 pellets were fired at 1250 °C/1300 °C and had higher C.C.S. than the Iron Ore No.2 pellets (Blaine 1989/2311 cm^2^/g) due to Ostwald ripening effect. Iron Ore No.2 ore has gangue content compared to Iron Ore No.1 mines, as a high Al_2_O_3_/SiO_3_ ratio influences the sintering of iron oxide grains that make the viscous melt, enhance pore size irregularity, and affect the pellet strength.
➢ H.P.G.D. is high in the microstructure of Iron Ore No.1 and No.2 pellets under 200 mesh, but beyond 200 mesh decreases due to increment of finer particle proportion. At temperate 1300 °C, S.P.G.D. is higher for 200 mesh for both Iron Ore No.1 and No.2 pellets. Blaine increases from 1312–1678 cm^2^/g for Iron Ore No.1 and 1989–2311 cm^2^/g for Iron Ore No.2. A decrease in P.P.G.D. shows proper sintering of oxides, which enhance the strength of pellets.
➢ XRD analysis of Iron Ore No.1 and No.2 pellets correspond to 200 mesh for different firings, concluding that iron oxide phases and ferrite phases (calcium and magnesium) directly influence the C.C.S. and AP. As the firing temperature 1250 °C/1300 °C/1350 °C, hematite phase percentage 71.1%, 61.1%, and 57%; magnetite phase 6.1%, 6%, and 3% decreases, while ferrite phases 22.2%, 26%, and 35% increase for Iron Ore No.1 pellet. For Iron Ore No.2, pellet hematite phase percentage varies from 61% to 55% and increases to 67%; magnetite phase varies from 9% to 18% and decreases to 6%, while ferrite phases increase from 21% to 27%, and up to 28.8%.
➢ Firing at 1350 °C has a high H.P.G.D. and shows high R.D.I. compared to pellets firing at 1300 °C/1250 °C with low R.D.I. Pore phase density is high for the Iron Ore No.2 pellet, at 1350 °C rather than 1300 °C. If phase percentage is high, it shows high R.D.I. for pellets fired at 1250 °C. Iron Ore No.1 pellets have coarser fines compared to Iron Ore No.2 pellets, which show good apparent porosity (A.P.) and offer good R.I. The pellet fired at 1250 °C reflects the higher reducibility, since they have a lower magnetite phase and less silicate/slag bond with a higher pore phase. Pellets fired at 1350 °C have more slag bonding with a reduced pore phase, less density, and more magnetite grains, thus showing lower reducibility.
➢ Chemical bonds in phases reflected in IR Spectroscopy show iron oxides phase 471.59 cm^−1^ and 550.18 cm^−1^ for K2B, as well as phase peaks 472.27 cm^−1^ and 550.44 cm^−1^ for M2B. Here, the broader range of peaks with high intensity reflects the bond affiliation of their dominant presence.
➢ For high activation energy, hematite dissociation reaction rate dependence at temperature should be high. As compared to Iron Ore No.2, the sample has high activation energy corresponding to the same heating rate of Iron Ore No.1. Due to the fineness of the ore particles and gangue content, it is higher for Iron Ore No.2 fines compared to Iron Ore No.1 fines.

## Figures and Tables

**Table 1:** Size fraction of (Iron Ore No.1) iron ore used size (µm).

Blaine No. (cm^2^/g)	+210	−210 + 149	−149 + 105	−105 + 74	−74 + 53	−53 + 44	−44
1312	31.2	12.1	7.2	5	2	2.9	39.6
1678	23.6	11	6.4	6.5	3.1	3.4	46
2425	11.4	9.1	7	6.2	6	4.2	56.1
3218	6	3.1	5.7	6.1	5.8	4.3	69

**Table 2:** Size fraction of (Iron Ore No.2) iron ore used size (µm).

Blaine No. (cm^2^/g)	+210	−210 + 149	−149 + 105	−105 + 74	−74 + 53	−53 + 44	−44
1989	19.2	11.7	6.1	5.6	4.1	5.1	48.2
2311	17.2	10.2	6.2	5.2	3.1	4.9	53.2
2879	9	8.1	6	6.1	2.8	3	65
3461	7	3.4	3.1	2.7	2.3	3.5	78

**Table 3:** Prepared Samples by using different blain fineness and firing temperatures.

No.	Mesh No.	Firing Temperature (°C)	Sample-Code IO No.1	Blain No. IO No.1	Sample-Code IO No.2	Blain No. IO No.2
**1**	100	1250	**K1A**	1312	**M1A**	1989
**2**	100	1300	**K1B**	1312	**M1B**	1989
**3**	100	1350	**K1C**	1312	**M1C**	1989
**4**	200	1250	**K2A**	1678	**M2A**	2311
**5**	200	1300	**K2B**	1678	**M2B**	2311
**6**	200	1350	**K2C**	1678	**M2C**	2311
**7**	300	1250	**K3A**	2425	**M3A**	2879
**8**	300	1300	**K3B**	2425	**M3B**	2879
**9**	300	1350	**K3C**	2425	**M3C**	2879
**10**	400	1250	**K4A**	3218	**M4A**	3461
**11**	400	1300	**K4B**	3218	**M4B**	3461
**12**	400	1350	**K4C**	3218	**M4C**	3461

**Table 4:** EDS analysis.

PHASE	Wt.%	Pellet (a)	Pellet (b)	Pellet (c)	Pellet (d)	Pellet (e)	Pellet (f)
		K2A	K2B	K2C	M2A	M2B	M2C
A	Fe	64.59	66.94	61.26	60.15	62.03	59.54
	O	28.45	32.06	29.88	30.02	29.34	35.04
	Al	1.13	1.06	1.07	0.95	2.21	2.58
	Si	1.11	0	0.27	0.61	0.8	0.74
B	O	27.84	36.42	41.04	41.57	41.48	47.29
	Si	0.39	4.35	17.35	16.79	3.96	17.14
	Ca	0.6	4.35	16.14	13.29	4.33	11.86
	Fe	6.92	17.19	14.17	7.25	19.09	11.97
	Al	0	24.2	6.01	17.42	17.25	10.96
	Mg	0	3.53	3.66	1.05	5.46	1.6

**Table 5:** TGA Kinetics.

	**(a) Iron Ore No.1**		
Heating Rate (°C/min)	Slope	R	Activation Energy (kJ/mol)
5	−1.67504	8.314	32.07223
10	−1.69985	8.314	32.54727
15	−1.74195	8.314	33.35336
	**(b) Iron Ore No.2**		
Heating Rate (°C/min)	Slope	R	Activation Energy (kJ/mol)
5	−1.75224	8.314	33.55038
10	−1.76337	8.314	33.76349
15	−1.77891	8.314	34.06104
